# Formulation, optimization and characterization of allantoin-loaded chitosan nanoparticles to alleviate ethanol-induced gastric ulcer: in-vitro and in-vivo studies

**DOI:** 10.1038/s41598-021-81183-x

**Published:** 2021-01-26

**Authors:** Reham Mokhtar Aman, Randa A. Zaghloul, Marwa S. El-Dahhan

**Affiliations:** 1 Department of Pharmaceutics, Faculty of Pharmacy, Mansoura University, Mansoura, 35516 Egypt; 2 Department of Biochemistry, Faculty of Pharmacy, Mansoura University, Mansoura, 35516 Egypt

**Keywords:** Biomarkers, Nanoscience and technology

## Abstract

Allantoin (ALL) is a phytochemical possessing an impressive array of biological activities. Nonetheless, developing a nanostructured delivery system targeted to augment the gastric antiulcerogenic activity of ALL has not been so far investigated. Consequently, in this survey, ALL-loaded chitosan/sodium tripolyphosphate nanoparticles (ALL-loaded CS/STPP NPs) were prepared by ionotropic gelation technique and thoroughly characterized. A full 2^4^ factorial design was adopted using four independently controlled parameters (ICPs). Comprehensive characterization, in vitro evaluations as well as antiulcerogenic activity study against ethanol-induced gastric ulcer in rats of the optimized NPs formula were conducted. The optimized NPs formula, (CS (1.5% w/v), STPP (0.3% w/v), CS:STPP volume ratio (5:1), ALL amount (13 mg)), was the most convenient one with drug content of 6.26 mg, drug entrapment efficiency % of 48.12%, particle size of 508.3 nm, polydispersity index 0.29 and ζ-potential of + 35.70 mV. It displayed a sustained in vitro release profile and mucoadhesive strength of 45.55%. ALL-loaded CS/STPP NPs (F-9) provoked remarkable antiulcerogenic activity against ethanol-induced gastric ulceration in rats, which was accentuated by histopathological, immunohistochemical (IHC) and biochemical studies. In conclusion, the prepared ALL-loaded CS/STPP NPs could be presented to the phytomedicine field as an auspicious oral delivery system for gastric ulceration management.

## Introduction

Nanotechnology-based approaches, involving biologically active phytochemicals, have awarded several innovational delivery systems; including polymeric nanoparticles (PNPs). Oral PNPs are auspicious drug carriers owing to their nanoscopic dimensions, targetability, bioadhesion, and controlled release of drugs in the gastrointestinal tract (GIT), therefore imparting improved bioavailability. The adherence ability of these PNPs to the mucosa and/or their ability to cross the mucosal barrier directly represents an imperative step before the translocation process of particles. From such standpoint, bioadhesion has a pivotal role to play in delivering the loaded drugs across the epithelia, averting presystemic metabolism as well as enzymatic degradation in the GIT^1,2^.

Amongst naturally occurring mucoadhesive cationic polysaccharides, chitosan (CS) has been extensively studied for PNPs preparation owing to its characteristic features. Low molecular weight (low MW) CS shows enhanced biocompatibility, biodegradability, solubility and less toxicity compared to high MW CS^3,4^. Ionotropic gelation method, an easy and attractive physical cross-linking process, is a cost-effective technique adopted to prepare chitosan nanoparticles (CS-NPs). This technique depends on ionic interactions arise from electrostatic attraction between two groups of opposite charge namely; positive amino groups (–NH3^+^) of CS as well as negative phosphate groups of sodium tripolyphosphate (STPP). Such combination was asserted to be non-toxic and hindered the possibility of drug degradation. Moreover, CS-NPs are positively-charged with mucoadhesive as well as permeation enhancing properties, thus facilitate opening of the epithelial tight junctions^2–4^.

Gastric ulcer, being one of the most widespread GI inflammatory diseases, affects approximately 5–10% of people worldwide. Over the decades, the inception of gastric ulcer is mainly pertained to the disturbance of the balance between endogenous aggressive factors (overproduction of hydrochloric acid and pepsin, helicobacter pylori) and endogenous defensive factors (mucin, bicarbonate, antioxidants, prostaglandins (PGs), nitric oxide and growth factors) in the gastric mucosa. Different drug categories are available as gastric ulcer therapies such as antacids, anti-cholinergic drugs, proton-pump inhibitors, and histamine H_2_-receptor antagonists. However, several side effects, limited efficacy and elevated incidence of recurrence limited their application and subsequently motivated the search for new safe and effective anti-ulcer therapies. Since gastric ulceration is a multi-etiological disorder, identification of biologically active phytochemicals that possess natural anti-ulcer properties such as the ability to reinforce the defensive endogenous capacity of the gastric mucosa, besides reduction of inflammation and gastric acid secretion may be beneficial in the amelioration of gastric ulcers^2,5^.

**Figure 1:**
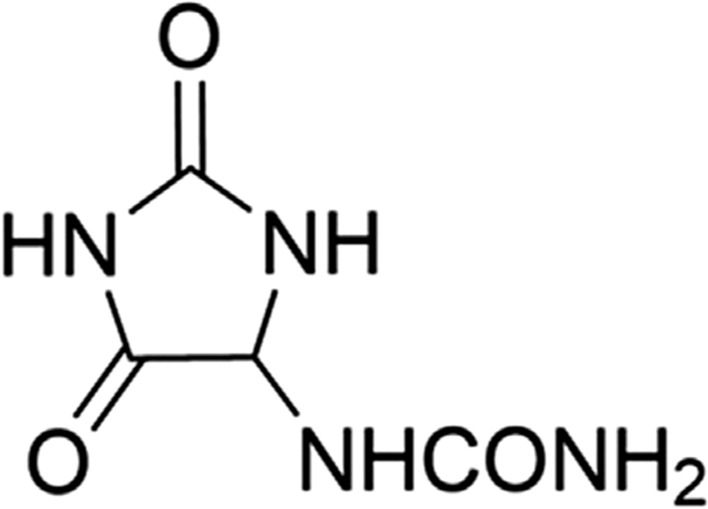
Chemical structure of ALL.

Allantoin (ALL) (Fig. 1) ((2,5-Dioxo-4-imidazolidinyl) urea) is a biologically active phytochemical extracted from the roots of *Symphytum officinale* (comfrey) native to Europe^6^. It possesses wound healing and tissue regeneration activities, besides renowned anti-oxidant, anti-inflammatory, pain reducing and gastroprotective effects that have been proved using a diversity of in vitro assay methods, in vivo animal models as well as clinical studies^7–12^.

Despite having multilateral pharmacological activities, little trials to fabricate and evaluate ALL in different delivery systems were stated. An earlier study was performed to improve the antifungal activity of copaiba oil, via the development of innovative solid lipid nanoparticles besides incorporation of ALL in the formulation, as well^13^. ALL loaded porous silica nanoparticles (PSNs) immobilized polycaprolacton (PCL) biocompatible nanocomposites (PCL/PSNs) were also prepared and characterized for biomedical applications^14^. Another study was implemented to investigate the performance of conventional as well as new argan oil enriched liposomes containing ALL as dermal drug delivery systems^15^. Recently, ALL was incorporated into CS membranes to evaluate its wound healing and tissue regeneration properties^16^. Consequently, different drug delivery systems (DDSs) are still required to be constructed and evaluated to potentiate and exploit the multilateral pharmacological bioactivities of ALL.

Although documented lines of substantiation manifesting the gastroprotective activity of ALL against common agents which attack the gastric mucosa, such as nonsteroidal anti-inflammatory drug (NSAID), stress, and ethanol, fabricating a nanostructured delivery system targeted at augmenting the gastroprotective activity of ALL has not been investigated as yet.

Hence, such survey lays the groundwork to dedicate the current study to prepare an oral phyto-pharmaceutical nanoparticulate system loaded with ALL (based on the cross-linking phenomenon between CS and STPP) to explore its prospective for effective gastric antiulcerogenic activity (AA).

Optimization of ALL-loaded chitosan/sodium tripolyphosphate nanoparticles (ALL-loaded CS/STPP NPs) was performed by espousing 2^4^ fully crossed design with four independently controlled parameters (ICPs) at two levels. The concentration of both CS (X_1_) and STPP (X_2_), CS:STPP volume ratio (X_3_) and amount of ALL (X_4_) were the different ICPs. The dependently measured parameters (DMPs) were drug content (Q), drug entrapment efficiency % (DEE %), particle size and ζ-potential (ZP) of ALL-loaded CS/STPP NPs. The optimized ALL-loaded CS/STPP NPs formula would be extensively investigated and further characterized with regard to its in vitro release, physical stability, and finally in vivo gastric AA in rats.

## Materials and methods

### Materials

ALL, STPP, omeprazole (ome) and mucin from porcine stomach were procured from Sigma-Aldrich (Saint Louis, MO, USA). Low MW CS was kindely provided by Primex (ChitoClear HQG 10, Code No.: 43000, Batch No.: TM4870, deacetylation degree 95%, Siglufjörður, Iceland). Analytical grade of absolute ethanol, glacial acetic acid (99%), sodium hydroxide (NaOH), and sodium carboxymethylcellulose (sodium CMC) were purchased from El-Nasr Pharmaceutical Chemical Co., Cairo, Egypt. The enzyme-linked immunosorbent assay (ELISA) kit of Interleukin-6 (IL-6) was purchased from Promokine Co., Heidelberg, Germany. Nitric oxide (NO) and oxidative stress markers assay kits were procured from Biodiagnostic co., Dokki, Giza, Egypt. Nuclear factor erythroid 2-related factor-2 (Nrf-2) and tumor necrosis factor-α (TNF-α) antibodies were purchased from Novus Biologicals, Centennial, CO 80112, USA.

### Design of experiment (DoE)

DoE was challenged as an organized and structured technique for optimizing the ionotropic gelation method. It provides very accurate as well as precise analysis for the interactions between the input factors affecting the output responses, through the establishment of mathematical models^17^. Fully crossed design (2^4^), as an experimental approach, allows the investigation of the individual effects and interactions of ICPs namely; CS concentration (X_1_), STPP concentration (X_2_), CS:STPP volume ratio (X_3_) and ALL amount (X_4_) on different DMPs such as Q, DEE %, particle size and ZP of ALL-loaded CS/STPP NPs. Two levels for each of the four ICPs were experimented and symbolized by the coded factor levels as + 1 (high levels) and − 1 (low levels) as presented in Table 1. Such levels were chosen based on preliminary studies and the optimization procedure was established within these domains. Three replicates were conducted for each combination, total number of sixteen formulations, to prepare ALL-loaded CS/STPP NPs (Table 2).

**Table 1:** ICPs and levels of a 2^4^ full factorial design.

ICPs	Maximum coded level	Minimum coded level	Maximum level	Minimum level
(X_1_) CS concentration (%, w/v)	+ 1	− 1	1.5	1
(X_2_) STPP concentration (%, w/v)	+ 1	− 1	0.5	0.3
(X_3_) CS:STPP volume ratio	+ 1	− 1	5:1	3:1
(X_4_) ALL amount (mg)	+ 1	− 1	13	10

**Table 2:** Formulations and properties of ALL-loaded CS/STPP NPs prepared according to 2^4^ full factorial design.

Formula code	Coded levels of				Q (mg)	DEE %	PDI	Particle size (nm)	ZP (mV)
	X_1_	X_2_	X_3_	X_4_					
F-1	−	+	+	−	4.91 ± 0.04	49.08 ± 0.39	0.178 ± 0.04	610.97 ± 17.01	22.97 ± 0.98
F-2	−	+	−	−	3.51 ± 0.05	35.14 ± 0.54	0.264 ± 0.01	665.27 ± 18.85	24.83 ± 0.60
F-3	+	+	+	−	2.91 ± 0.25	29.11 ± 2.53	0.116 ± 0.04	676.13 ± 15.21	34.53 ± 2.31
F-4	+	+	−	−	3.16 ± 0.04	31.58 ± 0.41	0.171 ± 0.04	747.53 ± 33.22	29.90 ± 0.62
F-5	+	−	+	−	5.35 ± 0.18	53.51 ± 1.77	0.212 ± 0.05	681.03 ± 20.57	29.63 ± 1.06
F-6	+	−	−	−	4.51 ± 0.26	45.12 ± 2.58	0.282 ± 0.04	735.70 ± 2.25	29.33 ± 1.15
F-7	−	−	+	−	3.60 ± 0.09	36.02 ± 0.93	0.230 ± 0.04	465.07 ± 20.51	28.40 ± 1.44
F-8	−	−	−	−	4.69 ± 0.18	46.85 ± 1.85	0.251 ± 0.06	507.90 ± 13.25	28.33 ± 0.21
F-9	+	−	+	+	6.26 ± 0.03	48.12 ± 0.21	0.290 ± 0.02	508.30 ± 13.35	35.70 ± 0.82
F-10	+	−	−	+	5.12 ± 0.07	39.40 ± 0.58	0.346 ± 0.01	512.73 ± 40.75	29.47 ± 0.64
F-11	−	−	+	+	3.03 ± 0.29	23.34 ± 2.27	0.320 ± 0.06	479.07 ± 7.53	28.57 ± 0.42
F-12	−	−	−	+	4.20 ± 0.13	32.34 ± 1.01	0.389 ± 0.08	493.50 ± 15.81	27.47 ± 0.57
F-13	−	+	−	+	1.87 ± 0.07	14.42 ± 0.55	0.478 ± 0.04	604.37 ± 13.24	28.33 ± 0.91
F-14	+	+	−	+	4.30 ± 0.42	32.93 ± 2.99	0.281 ± 0.05	791.67 ± 27.37	34.27 ± 0.93
F-15	−	+	+	+	3.81 ± 0.24	29.33 ± 1.86	0.413 ± 0.09	478.13 ± 15.76	28.27 ± 0.75
F-16	+	+	+	+	5.22 ± 0.08	40.18 ± 0.61	0.235 ± 0.03	711.43 ± 16.83	35.80 ± 0.92

Each value represents the mean ± SD (n = 3). X_1_, X_2_, X_3_, X_4_ are CS concentration, STPP concentration, CS:STPP volume ratio and ALL amount, respectively.

The complete polynomial regression equation was created as follows "Eq. (1)":

1 $$\begin{aligned} {\text{Y }} & = \upbeta_{{{0}}} + \, \upbeta_{{1}} {\text{X}}_{{1}} + \, \upbeta_{{2}} {\text{X}}_{{2}} + \, \upbeta_{{3}} {\text{X}}_{{3}} + \upbeta_{{4}} {\text{X}}_{{4}} + \upbeta_{{5}} {\text{X}}_{{1}} {\text{X}}_{{2}} \hfill \\ & \quad + \upbeta_{{6}} {\text{X}}_{{1}} {\text{X}}_{{3}} + \upbeta_{{7}} {\text{X}}_{{1}} {\text{X}}_{{4}} + \upbeta_{{8}} {\text{X}}_{{2}} {\text{X}}_{{3}} + \upbeta_{{9}} {\text{X}}_{{2}} {\text{X}}_{{4}} + \upbeta_{{{1}0}} {\text{X}}_{{3}} {\text{X}}_{{4}} \hfill \\ & \quad + \upbeta_{{{11}}} {\text{X}}_{{1}} {\text{X}}_{{2}} {\text{X}}_{{3}} + \upbeta_{{{12}}} {\text{X}}_{{1}} {\text{X}}_{{2}} {\text{X}}_{{4}} + \upbeta_{{{13}}} {\text{X}}_{{1}} {\text{X}}_{{3}} {\text{X}}_{{4}} + \upbeta_{{{14}}} {\text{X}}_{{2}} {\text{X}}_{{3}} {\text{X}}_{{4}} + \upbeta_{{{15}}} {\text{X}}_{{1}} {\text{X}}_{{2}} {\text{X}}_{{3}} {\text{X}}_{{4}} \hfill \\ \end{aligned}$$

where Y: DMP, β_o_: the arithmetical mean response of the 16 runs, β_1_, β_2_, β_3_, and β_4_: linear coefficients, β_5_, β_6_, β_7_, β_8_, β_9_ and β_10_: interaction coefficients between the two ICPs, β_11_, β_12_, β_13_, and β_14_: interaction coefficients between the three ICPs, Β_15_: interaction coefficient between the four ICPs, X_1_, X_2_, X_3_ and X_4_: ICPs.

### Preparation of ALL-loaded CS/STPP NPs

The ionotropic gelation method was followed in the preparation of ALL-loaded CS/STPP NPs, as previously described, which relies on the basis of an ionic interaction between the primary amino group of CS solution (positively-charged) and the phosphate group of STPP solution (negatively-charged)^18^. In brief, low MW CS (1 or 1.5% (w/v)) was dissolved in (1% v/v) aqueous acetic acid solution and magnetically stirred using magnetic stirrers (MS300HS, MTOPS Corp., Korea) until complete dissolution. CS's solution pH was elevated to 6 using 1 N NaOH, where such pH value maintains CS in its soluble form at the inspected concentrations^19^. Next, STPP aqueous solution was prepared at two different concentrations (0.3 or 0.5% (w/v)). Both CS as well as STPP solutions were filtered through a 0.45 μm pore size filter (EMD Millipore, Billerica, MA, USA). The ALL-loaded CS/STPP NPs were formed spontaneously by the dropwise addition of STPP solution (containing an accurately weighed and completely dissolved quantity of ALL (10 or 13 mg)) onto CS solution at two different volume ratios (3:1 or 5:1) of CS and STPP, respectively. The dropping rate, using a disposable insulin needle, was 0.14 mL/min under constant magnetic stirring (1200 rpm) at room temperature. Further cross-linking reaction was allowed to proceed with continous stirring for 30 min. Then, the ALL-loaded CS/STPP NPs were separated from the unentrapped ALL by centrifugation at 13,000 rpm under cooling conditions at 4 °C for 4 h (CE16-4X100RD, ACCULAB, USA), washed with deionized water (DW), resuspended in DW, and then freeze-dried (SIM FD8-8T, SIM international, USA). Finally, the freeze-dried ALL-loaded CS/STPP NPs were kept at 4 °C for additional characterization. The clear supernatant containing unentrapped ALL was kept for estimation of efficiency of drug entrapment as percentage (DEE %). For preparation of plain CS/STPP NPs corresponding to each formula, the same procedure was followed using STPP solutions without ALL.

### Characterization of ALL-loaded CS/STPP NPs

All the NPs of the 16 formulae were subjected to appraisal of DMPs in terms of Q, DEE %, particle size, polydispersity index (PDI) and ZP.

#### Estimation of drug content

Actual drug content (Q) was assessed by quantifying the amount of unentrapped ALL in the clear supernatant of all medicated formulae after centrifugation at 13,000 rpm for 4 h at 4 °C. The content of the unentrapped ALL was measured spectrophotometrically, against the supernatant of each corresponding plain CS/STPP NPs as a blank, at λ_max_ 215 nm (ultraviolet/visible (UV–VIS) double beam spectrophotometer, Labomed Inc., USA).

The Q of ALL was indirectly determined by subtracting the amount of free ALL in the supernatant from the total amount of drug added initially according to Eq. (2):

2 $${\text{Q }} = {\text{ W}}_{{\text{t}}} - {\text{ W}}_{{\text{f}}}$$

where, W_t_ symbolizes the total amount of ALL available in the formulation and W_f_ symbolizes the amount of free ALL in the supernatant.

#### Determination of drug entrapment efficiency percent

Efficiency of drug entrapment as percentage (DEE %) was estimated for each formula according to the following Eq. (3):

3 $${\text{DEE }}\% = \frac{{{\text{Actual}}\;{\text{drug}}\;{\text{content}}\;\left( {\text{Q}} \right)}}{{{\text{Total}}\;{\text{drug}}\;{\text{content}}}} \times 100$$

#### Particle size measurements

The average hydrodynamic size as well as PDI of all the freshly prepared ALL-loaded CS/STPP NPs, after appropriate dilution with DW, were determined in triplicates by Zetasizer Nano ZS (Malvern Instruments, Malvern, UK) adopting the dynamic light scattering (DLS) mechanism.

#### ζ-potential (ZP)

ZP is an imperative factor to assess the colloidal dispersion stability. ZP measurement was carried out in DW utilizing Zetasizer Nano ZS (Malvern Instruments, Malvern, UK) adopting the laser Doppler micro-electrophoresis technique which observes the electrophoretic mobility of the NPs in an electrical field. All measurements were carried out in triplicates.

### Selection of the optimized formulation on the basis of desirability function

Using the desirability approach, numerical optimization was employed to locate the optimal levels of the ICPs to obtain the desired DMPs. Optimization was performed to obtain the levels of X_1_–X_4_ which keep particle size within the range of the obtained response, while maximize Q, DEE % and ZP. Optimized formulation (F-9) was selected on the predetermined basis, as well as with good desirability^20^.

### Evaluation of formula 9 (F-9) of ALL-loaded CS/STPP NPs

#### Morphology

The morphology of the optimized formula (F-9), freshly prepared, was visualized using transmission electron microscopy (TEM) (JEOL JEM-2100, JEOL Ltd., Tokyo, Japan). One mL of the NPs dispersion was suitably diluted with DW, sonicated for 5 min, cast onto carbon coated copper grid, the excess sample was wiped away using a filter paper, and finally the grid was air-dried at room temperature. Then, it was directly inspected via TEM without staining at 160 kV. The image was captured and the analysis process was carried out using imaging viewer software (Gatan Microscopy Suite Software, version 2.11.1404.0).

#### Fourier-transform infrared spectroscopy (FT-IR)

The infrared spectra of ALL, CS, STPP, and the corresponding physical mixture of the optimized formula besides lyophilized plain and medicated CS/STPP NPs (F-9), were traced using FT-IR Spectrophotometer (Madison Instruments, Middleton, WI, USA). Samples were homogeneously mixed with potassium bromide (KBr), compressed into discs and individually scanned over a wave number range of 500–4000 cm^−1^.

#### Thermal properties

Thermodynamic techniques were applied to reveal the heat stresses of pharmaceutical preparations, additives, besides their interactions during the process of formularization. Thermograms of ALL, CS, STPP, and the corresponding physical mixture of the optimized NPs formula as well as lyophilized plain and medicated CS/STPP NPs (F-9), were determined via differential scanning calorimetry (DSC) (DSC-60, Shimadzu Corporation, Japan) equipped with a Shimadzu TA-60 Series data processor which allow automatic display of data curves. Four milligrams (per each sample) were separately get heated, 10 °C/min as a heating rate, in hermetically sealed aluminum pans over a temperature range of 30–400 °C under a constant purging of dry nitrogen at 20 mL/min. Indium (purity of 99.99% and melting point of 156.6 °C), as a reference standard, was used to calibrate DSC runs.

#### Powder x-ray diffraction (PXRD)

Powder x-ray diffraction (PXRD) technique is a remarkable one in inspecting the changes in the compounds' crystallinity throughout the formulation process. PXRD patterns of ALL, CS, STPP, and the corresponding physical mixture of the optimized NPs formula as well as lyophilized plain and medicated CS/STPP NPs (F-9), were recorded at a scanning range from 3° to 50° at 2θ angle employing a Diano X-ray diffractometer (USA) equipped with Co-Kα radiation. To perform such analysis process, the used current and voltage were 9 mA and 45 kV, respectively.

#### In vitro ALL release study from ALL-loaded CS/STPP NPs (F-9)

The in vitro release profile of ALL from the freshly prepared medicated NPs (F-9), in comparison to its diffusion from aqueous solution, as a control as previously reported, was adopted using locally fabricated vertical Franz diffusion cells with diffusional surface area of 7.07 cm^2,17^. Semipermeable SpectraPor dialysis membranes (Molecular weights cut off: 12,000–14,000 Da, Spectrum Medical Industries Inc., Los Angeles 90054, USA), that were equilibrated overnight either with 0.1 M HCl (pH 1.2), phosphate buffer (pH 6.8) or phosphate buffer (pH 7.4) as simulated gastrointestinal fluids before mounting in the diffusion cell, were tightly fixed between the donor and receptor compartments.

Briefly, ALL-loaded CS/STPP NPs (F-9), containing an equivalent amount of 12 mg ALL, were suspended in distilled water and placed in the donor compartment, while 50 mL of the dialysis medium was introduced to the receptor one. Throughout the whole experiment, the entire congregation of the used Franz diffusion cells was shaken by thermostatically controlled shaking incubator (GFL Gesellschaft für Labortechnik, Burgwedel, Germany) at 100 rpm/min and maintained at 37 ± 0.5 °C. At preplanned time intervals, 0.5, 1, 1.5, 2, 3, 4, 6, 8, 24, 32, 48 and 56 h, withdrawal of aliquots (3 mL) from the receptor medium was carried out followed by resubstitution with an equivalent volume of fresh medium, equilibrated at 37 ± 0.5 °C, in order to conserve a constant volume during the experiment. The assembled aliquots were filtered through 0.45 μm membrane filters and quantified spectrophotometrically for drug concentration using UV–VIS spectrophotometer, against the corresponding plain CS/STPP NPs that were treated similarly as the medicated.

Finally, the cumulative ALL released (%) was calculated at each time interval as an average value from three experiments. Contemporary, aqueous solution containing the same amount of ALL was also experimented for the process of diffusion similarly in triplicate.

#### Release kinetic

To attain a profound knowledge about the mechanism of drug release from the NPs, the in vitro release data of the selected ALL-loaded CS/STPP NPs (F-9) were fitted to different kinetic models including zero-order, first-order as well as diffusion-controlled release^21^.

To verify the release mechanism, Korsmeyer–Peppas kinetic model was also applied as a logarithmic relation of the drug released fraction (M_t_/M_∞_) and the release time (t) "Eq. (4)":

4 $$({\text{M}}_{{\text{t}}} /{\text{M}}_{\infty } = {\text{ kt}}^{{\text{n}}} )$$

where n is the characteristic diffusional exponent for the release mechanism calculated as the slope of the plot and k is the kinetic constant^22^. Moreover, the release data were fitted to the following Weibull kinetic model which is generally applied to systems prepared with swellable materials such as CS "Eq. (5)":

5 $$\left( {{\text{ln}}\left[ { - {\text{ ln}}\left( {{1 } - {\text{ F}}} \right)} \right] = \upbeta {\text{ ln td }} + \upbeta {\text{ ln t}}} \right)$$

where F and td stand for the fraction of amount drug released up to time t and the lag time before the drug release takes place, respectively, while β characterizes the shape of the release curve^23^. The model which gives a coefficient of determination (R^2^) close to 1 would be considered as the order of release.

#### Mucoadhesive strength

The mucoadhesive properties of ALL-loaded CS/STPP NPs (F-9) were investigated through incubation of these NPs with porcine mucin. Two in vitro methods were used in such study.

### Determination of mucin-binding efficiency (%)

This method was based on assessing the NPs adherence to the mucosa by studying the interaction between the negatively-charged mucin (as the mucosal component) and the positively-charged CS/STPP NPs in aqueous solution^2,24–26^. Briefly, 5 mL of both reconstituted NPs (F-9 aqueous dispersion) and mucin (0.5 mg/mL in phosphate buffer saline (PBS) pH 7.4) were vortexed (Model VM-300, Gemmy Industrial Corp., Taiwan), incubated at 37 °C for 1 h and subsequently centrifuged at 10,000 rpm for 1 h. The amount of free mucin in the supernatant was determined at 253 nm by UV–VIS spectrophotometer. This experiment was carried out in triplicate and the average mucin-binding efficiency (%), expressing the mucoadhesive strength of the CS/STPP NPs, was estimated according to the following Eq. (6):

6 $${\text{Mucin-binding efficiency }}(\% ) = \frac{\text{Total amount of mucin} - {\text{free amount of mucin}}}{\text{Total amount of mucin}} \times 100$$

### Determination of ZP of CS/STPP-mucin mixtures

Such method was employed to evaluate the influence of mucin on the ZP of CS/STPP NPs. ZP of ALL-loaded CS/STPP NPs (F-9 aqueous dispersion) after incubation with mucin, as previously described, was determined. Moreover, equal volumes of mucin and CS (5 mL each) were vortexed, incubated at 37 °C for 1 h and subsequently the ZP of such mixture was measured. Besides, ZP of both mucin and CS solutions were evaluated as well^27–29^.

#### Short-term physical stability of ALL-loaded CS/STPP NPs (F-9)

The influence of storage conditions (namely; temperature) on the physical stability of the optimized ALL-loaded CS/STPP NPs (F-9) was assessed. The freshly prepared ALL-loaded CS/STPP NPs dispersions (F-9) were filled in screw capped glass bottles and stored at refrigeration (4 ± 1 °C) and ambient conditions for 3 months without any stirring or agitation^30^. The NPs were assessed regarding particle size, PDI, ZP and average drug retention (%) at zero time (at production day as described above previously), and after 1 and 3 months of storage.

#### In vivo assessment studies

##### Animals

Animal protocol was revised and approved by the ethical committee of Faculty of Pharmacy, Mansoura University, Mansoura, Egypt, in accordance with “principles of laboratory animal care NIH publication revised 1985” (Code number: 2020–107). Moreover, the study was conducted and presented with careful consideration of the Animal Research: Reporting of In Vivo Experiments (ARRIVE) guidelines. Forty-two healthy male Sprague–Dawley rats, weighing between 180 and 200 g, were housed under standard conditions of temperature (25 °C ± 1) with a regular 12 h light/dark cycle and free access to standard animal chew.

### Evaluation of AA against ethanol-induced gastric ulcer in rats

Rats were kept for a week for acclimatization before being randomly divided into seven equal groups (n = 6). The groups received the followings for five consecutive days via intragastric tube;

- Group a: Normal control (N); rats received only water.
- Group b: Ulcer; rats received only water.
- Group c: Ome (rats received 20 mg/kg of ome suspended in sodium CMC (1% w/v)).
- Group d: Plain; rats received the nanocarrier (plain CS/STPP NPs, F-9).
- Group e: Pure drug (ALL); rats received 60 mg/kg of ALL aqueous solution (ALL-sol).
- Group f: Nano ALL low dose (NanoAL); rats received ALL-loaded CS/STPP NPs (F-9, 30 mg/kg).
- Group g: Nano ALL high dose (NanoAH); rats received ALL-loaded CS/STPP NPs (F-9, 60 mg/kg).

On the fifth day, 24 h-fasted rats received their last dose of treatment, and then an hour later all the groups, except for N group, were given a single dose of (5 mL/kg) of absolute ethanol via intragastric tube^31^. Plain and medicated CS/STPP NPs were prepared as previously mentioned. In the present study, ome and pure ALL were applied for comparison. Their doses were selected based on previous studies^32,33^.

### Biological samples collection and macroscopic examination of gastric ulceration

An hour later to ethanol administration, blood samples were collected from the retro-orbital venous plexus in plain collection tubes. Sera were obtained by the subsequent centrifugation of the samples at 3000 rpm at 4 °C for 5 min (Sigma 2-16P centrifuge, Sigma Co., USA) and stored at − 20 °C for serum IL-6 assessment. Rats were sacrificed and undergone immediate laparotomy. Stomachs were separated and opened along the greater curvature. Gastric contents were collected in clean glass tubes for measurement of pH through digital pH meter (Consort NV P-901 pH-meter, Belgium, Europe). Stomachs were washed with cold normal saline, blotted dry and pinned to be photographed for assessment of the area of the gastric damage (mm^2^) with Image J software (1.52a, NIH, USA).

The ulcer index (UI) as well as the protection index (PI) were evaluated according to Eqs. (7 and 8), respectively^34,35^:

7 $${\text{UI}} = \frac{{{\text{Total}}\;{\text{area}}\;{\text{of}}\;{\text{lesions}}}}{{{\text{Total}}\;{\text{area}}\;{\text{of}}\;{\text{stomach}}}} \times 100$$

8 $${\text{PI}} = \frac{{{\text{UI}}\;{\text{of}}\;{\text{ulcer}}\;{\text{group}} - {\text{UI}}\;{\text{of}}\;{\text{treated}}\;{\text{group}}}}{{{\text{UI}}\;{\text{of}}\;{\text{ulcer}}\;{\text{group}}}} \times 100$$

After the completion of the macroscopic examination, the glandular part of the stomach was then dissected into two portions. The first portion was preserved in 10% buffered formalin and embedded in paraffin wax for further histopathological and immunohistochemical (IHC) investigations. The second cut of the tissue was used to prepare 10% homogenate by the homogenization of the tissue in an ice-cold phosphate-buffered saline followed by a storage of the homogenate aliquots at − 80 °C for subsequent biochemical evaluations.

### Histopathological examination and IHC localization of Nrf-2 and TNF-α

Paraffin blocks of the formalin-preserved glandular part of the stomach were cut as 5 μm-thick sections. One set of sections was picked up on slides, deparaffinized, and stained with hematoxylin and eosin (H&E) as well as the special stain Alcian blue. Another set of sections, along their appropriate positive control sections, were processed for IHC staining using monoclonal antibodies for Nrf-2 and TNF-α. The procedure was as described in our previous study^36^. Slides were photographed using a digital camera-aided computer system (Nikon digital camera, Tokyo, Japan). Both examinations were performed by two qualified pathologists unaware of the specimens identity, in order to prevent any bias. The intensity of the staining was scored as follows: (−), negative; (+), weak; (++), moderate; and (+++), strong staining^37^. Both the oxidative stress markers, Gastric MDA and GSH, and the pro-inflammatory markers, gastric NO and serum IL-6, were assessed via the appropriate commercially available colorimetric and ELISA kits, according to the manufacturer's instructions.

#### Statistical data analysis

The in vitro besides in vivo data were expressed as mean ± standard deviation (SD) and mean ± standard error of the mean (SEM), respectively. The data were analyzed statistically using one-way analysis of variance (ANOVA) followed by Tukey–Kramer multiple comparisons test, for in vitro data, as well as ANOVA followed by Tukey’s post-hoc test or Student's t-test (unpaired t-test), for in vivo ones. In order to finalize such analysis process, GraphPad Prism version 5.00 (GraphPad Software, Inc., La Jolla, CA, USA) was used. The applied experimental approach (2^4^ fully crossed design) was estimated in terms of statistical significance utilizing ANOVA by Design-Expert version 12 (Stat-Ease, Inc., Minneapolis, Min-nesota, USA). Statistically significant F-values (*p* < 0.05) and adjusted coefficients of determination (adjusted R^2^) ranged from 0.8 to 1.0 were the standards for evaluation of the selected model, as previously reported^17,38^. Besides, the effect of ICPs on the DMPs was presented as contour plots as well as response surface plots created by setting the X_3_ and X_4_ factors at their low and high levels and changing X_1_ and X_2_ over the range used in the study.

## Results and discussion

### Preparation, characterization and optimization of ALL-loaded CS/STPP NPs

In the present study, ALL-loaded CS/STPP NPs were successfully prepared by ionotropic gelation method. The applicability of CS-based NPs particularly for mucosal routes of administration (oral, nasal, pulmonary or vaginal) has been reported^39^. Low MW CS with highest degree of deacetylation was selected to favor preparation of electropositive smaller sized CS/STPP NPs; as previously demonstrated^19^. Hence, to the best of our knowledge, this is the first attempt for encapsulation of ALL in CS/STPP NPs.

Moreover, for ameliorating the drug absorption and consequently its therapeutic performance, vital DMPs including small particle size and PDI (within the range of the obtained responses) as well as maximum Q, DEE % and ZP have to be taken into account. Fully crossed design offered very accurate and precise analysis, revealed the ICPs interactions and extensively used in several processes^17^.

### Drug content (Q) and drug entrapment efficiency % (DEE %)

These two DMPs were picked out as indicators of the reproducibility and efficiency of the proceeding technique (Table 2). Q and DEE % of ALL-loaded CS/STPP NPs ranged from 1.87 ± 0.07 to 6.26 ± 0.03 mg and 14.42 ± 0.55 to 53.51 ± 1.77%, respectively. The linear regression models for these two DMPs are represented as follows "Eqs. (9 and 10)":

9 $$\begin{aligned} {\text{Q}} & = + 4.15 + \, 04499{\text{X}}_{{1}} - \, 04415{\text{X}}_{{2}} + \, 0.{233}0{\text{X}}_{{3}} + \, 0.0{\text{742X}}_{{4}} \hfill \\ & \quad - 0.{\text{2643X}}_{{1}} {\text{X}}_{{2}} + \, 0.0{\text{981X}}_{{1}} {\text{X}}_{{3}} + \, 0.{\text{5472X}}_{{1}} {\text{X}}_{{4}} + \, 0.{268}0{\text{X}}_{{2}} {\text{X}}_{{3}} \hfill \\ & \quad + \, 0.0{\text{159X}}_{{2}} {\text{X}}_{{4}} + \, 0.{12}0{\text{2X}}_{{3}} {\text{X}}_{{4}} - \, 0.{43}00{\text{X}}_{{1}} {\text{X}}_{{2}} {\text{X}}_{{3}} + \, 0.{\text{2267X}}_{{1}} {\text{X}}_{{2}} {\text{X}}_{{4}} \hfill \\ & \quad + \, 0.0{\text{629X}}_{{1}} {\text{X}}_{{3}} {\text{X}}_{{4}} + \, 0.0{\text{943X}}_{{2}} {\text{X}}_{{3}} {\text{X}}_{{4}} + \, 0.0{\text{152X}}_{{1}} {\text{X}}_{{2}} {\text{X}}_{{3}} {\text{X}}_{{4}} \hfill \\ \end{aligned}$$

where F = 105.45, and *p* < 0.0001

10 $$\begin{aligned} {\text{DEE }}\% & = + { 36}.{66} + { 3}.{\text{339X}}_{{1}} - { 3}.{\text{933X}}_{{2}} + { 1}.{\text{932X}}_{{3}} - { 4}.{\text{146X}}_{{4}} \hfill \\& \quad - { 2}.{6}0{\text{9X}}_{{1}} {\text{X}}_{{2}} + \, 0.{8}0{\text{44X}}_{{1}} {\text{X}}_{{3}} + { 4}.{\text{312X}}_{{1}} {\text{X}}_{{4}} + { 2}.{\text{272X}}_{{2}} {\text{X}}_{{3}} \hfill \\ & \quad + \, 0.{641}0{\text{X}}_{{2}} {\text{X}}_{{4}} + \, 0.{8}0{\text{37X}}_{{3}} {\text{X}}_{{4}} - { 3}.{\text{812X}}_{{1}} {\text{X}}_{{2}} {\text{X}}_{{3}} + { 2}.{3}0{\text{1X}}_{{1}} {\text{X}}_{{2}} {\text{X}}_{{4}} \hfill \\ & \quad + \, 0.{\text{4523X}}_{{1}} {\text{X}}_{{3}} {\text{X}}_{{4}} + \, 0.{\text{5339X}}_{{2}} {\text{X}}_{{3}} {\text{X}}_{{4}} + \, 0.{64}0{\text{3X}}_{{1}} {\text{X}}_{{2}} {\text{X}}_{{3}} {\text{X}}_{{4}} \hfill \\ \end{aligned}$$

where F = 126.27, and *p* < 0.0001.

The quantitative effects of ICPs namely; CS concentration (X_1_), STPP concentration (X_2_), CS:STPP volume ratio (X_3_) as well as ALL amount (X_4_), and their interactions on the above DMPs are elucidated via the aforementioned equations. Circumspect examination of these two equations asserts that both ICPs namely; CS (X_1_) and CS:STPP (X_3_) have a favourable effect, while STPP (X_2_) has a reverse effect, on DMPs such as Q and DEE %. Interestingly, CS (X_1_) was the major factor affecting positively the above two responses. Presumably; STPP (X_2_), being the cross-linking agent, leads to water “expulsion” and promotes the “escape” of some of the dissolved drug molecules^40^. Both CS (X_1_) with its viscosity and CS:STPP (X_3_) with increased volume ratio synergistically antagonize this "escape” with a positive coefficient value of X_1_X_3_.

Table 2 depicts that an increment in CS (X_1_) from 1 to 1.5% w/v and keeping X_2_, X_3_ and X_4_ constant (F-5 and 7, F-9 and 11, besides F-13 and 14) increased Q and DEE % values, while an increase in STPP (X_2_) from 0.3 to 0.5% w/v and keeping X_1_, X_3_ and X4 constant (F-3 and 5, as well as F-12 and 13) decreased Q and DEE % values.

**Figure 2:**
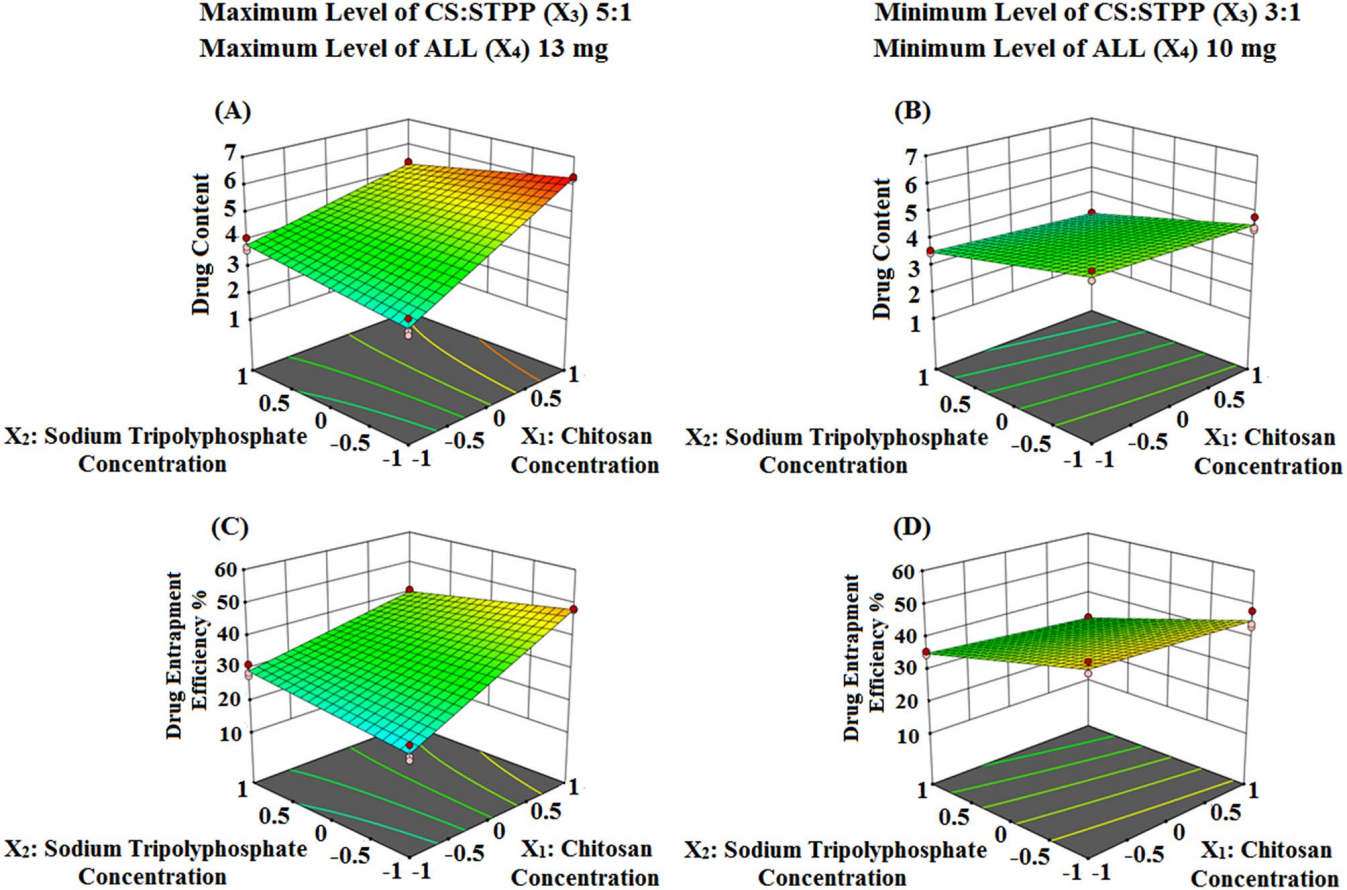
Three dimensional surface plots representing the effect of the interaction between the concentrations of both CS (X_1_) and STPP (X_2_) on Q and DEE % (**A** and **B**) and (**C** and **D**) at the maximum and minimum levels of both CS:STPP volume ratio (X_3_) and ALL amount (X_4_), respectively, using Design-Expert version 12 (/https://www.statease.com/software/design-expert).

**Figure 3:**
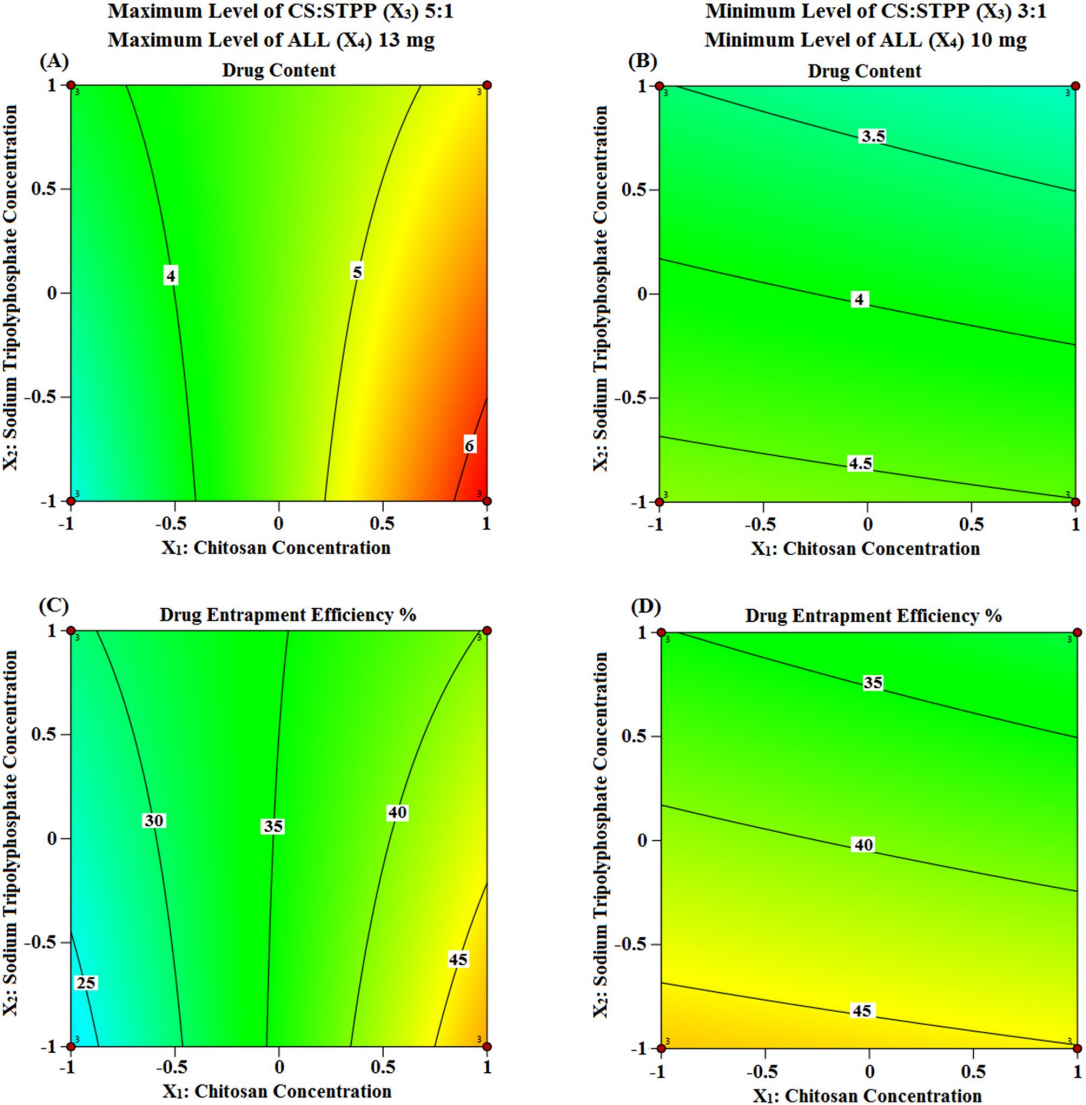
Contour plots representing the effect of the interaction between the concentrations of both CS (X_1_) and STPP (X_2_) on Q and DEE % (**A** and **B**) and (**C** and **D**) at the maximum and minimum levels of both CS:STPP volume ratio (X_3_) and ALL amount (X_4_), respectively, using Design-Expert version 12 (/https://www.statease.com/software/design-expert).

Nevertheless, to allow for much easier interpretation of ICPs influence on DMPs, various graphical plots were generated based on the model equations for Q and DEE %. Figures 2A–D and 3A–D show the response surface and contour plots, respectively, of variations in the aforementioned DMPs against two ICPs, X_1_ (CS) and X_2_ (STPP) at a time, keeping the other ICPs, X_3_ and X_4_ (CS:STPP and ALL), fixed at their high and low levels. The contour plots illustrate that the two DMPs exhibit highest Q (6.26 ± 0.03 mg) and high DEE % (48.12 ± 0.21%) values at high level of CS (X_1_) and low level of STPP (X_2_) when CS:STPP (X_3_) and ALL (X_4_) were at their high levels. This implies that the use of high levels of CS (X_1_), CS:STPP (X_3_), ALL (X_4_) and low level of STPP (X_2_), over the range used in the study, is required to prepare NPs with high Q and DEE % (F-9).

The prospective explication is that high concentration of CS (X_1_), with increased viscosity, minimizes the amount of drug departure from the NPs matrix, and consequently increases DEE % value. Additionally, high polymer concentration increases the formed CS/STPP NPs with subsequent increase in the amount of entrapped ALL. These results were in accordance with reported studies^41–43^.

### Particle size and the polydispersity index (PDI)

One of the foremost determiners in epithelial and mucosal tissue uptake of NPs, along with their intracellular bargaining, hence having an impact on the therapeutic efficacy is the particle size. Polymeric NPs ranged from 50 to 500 nm were reported to have maximal uptake and interaction with mucosal epithelial membranes^3^. Not merely the average particle size (nm) of NPs is prime, but also their size variability. PDI, also known as the heterogeneity index, is a dimensionless and scaled index derived from the cumulants analysis which describes the relative discrepancy of the size distribution of particles. PDI, based on its value ranging from 0 to 1, has an influence on the therapeutic performance and the pharmacokinetic parameters of the medicated NPs formulations. Both of which are two substantial indicias for estimating size stability of a colloidal dosage form upon storage^2,17^.

Particle size and PDI of the prepared ALL-loaded CS/STPP NPs are displayed in Table 2. All formulae exhibited a PDI values ranged from 0.116 ± 0.04 to 0.478 ± 0.04. The small values of PDI (< 0.5) depict narrow-average distribution and are referred to as a homogenic dispersion. Additionally, they are necessary to keep the stability of the colloidal dosage form without precipitates or microparticles formation^44^.

The linear regression model for particle size is symbolized as follows "Eq. (11)":

11 $$\begin{aligned} {\text{P size}} = & + 604.3 + 66.27{\text{X}}_{1} + 56.39{\text{X}}_{2} - 28.03{\text{X}}_{3} \\ & - 31.90{\text{X}}_{4} + 4.737{\text{X}}_{1} {\text{X}}_{2} + 1.692{\text{X}}_{1} {\text{X}}_{3} - 7.633{\text{X}}_{1} {\text{X}}_{4} \\ & - 13.49{\text{X}}_{2} {\text{X}}_{3} + 17.61{\text{X}}_{2} {\text{X}}_{4} - {\mkern 1mu} 0.1333{\text{X}}_{3} {\text{X}}_{4} + 1.921{\text{X}}_{1} {\text{X}}_{2} {\text{X}}_{3} \\ & + 41.78{\text{X}}_{1} {\text{X}}_{2} {\text{X}}_{4} + 5.308{\text{X}}_{1} {\text{X}}_{3} {\text{X}}_{4} - 9.962{\text{X}}_{2} {\text{X}}_{3} {\text{X}}_{4} + 2.579{\text{X}}_{1} {\text{X}}_{2} {\text{X}}_{3} {\text{X}}_{4} \\ \end{aligned}$$

where F = 91.72, and *p* < 0.0001.

Such equation depicts that particle size is substantially impacted by all ICPs and their interactions. The most conspicuous effect on particle size enlargement was attributed to the concentration of both CS (X_1_) and STPP (X_2_), if their levels were kept high. However, CS:STPP volume ratio (X_3_) and ALL amount (X_4_) have an adverse influence as previously reported^19,45^.

Moreover, circumspect inspection of Table 2 demonstrates that the interaction between CS (X_1_) and STPP (X_2_), while keeping CS:STPP (X_3_) and ALL (X_4_) constant either at their low or high levels, is synergistic towards particle size (F-4 and 8 as well as F-11 and 16).

The substantial increment might be ascribed to an increase in the number of CS chains per volume by increasing its concentration (X_1_), hence forming larger particles when stirred with the cross-linking agent, STPP. Furthermore, the cross-linking density between CS and STPP decreases, resulting in particle aggregation and larger particles formation. Likewise, high concentration of STPP (X_2_) motivates a faster cross-linking phenomenon which might account for particle size increment. These results are in accordance with those obtained by reported investigations^46,47^.

### ζ-potential

NPs' surface charge density, expressed as ZP, is a prime parameter which affects strongly their cellular uptake ability, mucoadhesive property, and stability. Conventional NPs (without surface modification) and negatively-charged ones can be readily opsonized and extensively cleared by the reticuloendothelial system (RES) in the blood stream. On the other hand, positively-charged NPs favour adhesion to the cell mucosa which are normally negatively-charged, hence can be used as mucoadhesive drug delivery systems. As a rule of thumb, absolute ZP values above + 30 mV or below − 30 mV provide very good stability in the dispersion medium^2,17,48^.

Herein, the ZP values of all the prepared formulations were positively-charged. Such a tendency could be attributed to the presence of freely ionized amino groups (–NH3^+^) on the surface of the CS/STPP NPs, therefore, leading to much more powerful electrostatic repulsion between NPs with subsequent enhanced effect on the stability of the nano-dispersions. These data are in line with reported study^19^.

The linear regression model for ZP is represented as follows "Eq. (12)":

12 $$\begin{aligned} {\text{ZP}} & = + { 29}.{74} + { 2}.{\text{592X}}_{{1}} + \, 0.{125}0{\text{X}}_{{2}} + \, 0.{\text{7458X}}_{{3}} + { 1}.{\text{246X}}_{{4}} \hfill \\& \quad + { 1}.{\text{171X}}_{{1}} {\text{X}}_{{2}} + \, 0.{\text{8417X}}_{{1}} {\text{X}}_{{3}} + \, 0.{\text{2333X}}_{{1}} {\text{X}}_{{4}} - \, 0.{\text{2167X}}_{{2}} {\text{X}}_{{3}} \hfill \\ & \quad + \, 0.{\text{5583X}}_{{2}} {\text{X}}_{{4}} + \, 0.{\text{3542X}}_{{3}} {\text{X}}_{{4}} + \, 0.{17}0{\text{8X}}_{{1}} {\text{X}}_{{2}} {\text{X}}_{{3}} \hfill \\ & \quad - 0.{\text{6292X}}_{{1}} {\text{X}}_{{2}} {\text{X}}_{{4}} + { 6}.0{\text{6278E}}-{\text{16X}}_{{1}} {\text{X}}_{{3}} {\text{X}}_{{4}} - \, 0.{\text{5167X}}_{{2}} {\text{X}}_{{3}} {\text{X}}_{{4}} - \, 0.{\text{6125X}}_{{1}} {\text{X}}_{{2}} {\text{X}}_{{3}} {\text{X}}_{{4}} \hfill \\ \end{aligned}$$

where F = 39.39, and *p* < 0.0001.

Consistently, positive ZP values for all the prepared formulae were obtained in the range of 22.97 ± 0.98 to 35.80 ± 0.92 (F-1 and 16, Table 2).

Positive ZP is known to be rudiment for bioavailability increment through mucoadhesion. The aforementioned equation shows that all the main ICPs have positive coefficient towards ZP. The final surface charge of CS/STPP NPs is considerably impacted by CS (X_1_), with the highest positive coefficient value, which defines the amount of available protonated amino groups (–NH3^+^) on the surface of NPs after preparation^19^. The statistical significance of all ICPs and their interactions on ZP and other DMPs is epitomized in Supplementary Table S1 online.

Besides, a Fit statistics summary for the linear regression analysis models of all the DMPs is depicted in Supplementary Table S2 online. High R-squared, Adjusted R-squared and Predicted R-squared values, ranged from 0.9486 to 0.9834, 0.9245 to 0.9756, and 0.8844 to 0.9626, respectively, indicate successful modeling of the results using the adopted design. Similarly, high values of adequate precision (> 4), ranged from 22.0360 to 42.4175, indicates the sufficiency of the model to navigate the space with the high signal-to-noise ratio of the results.

### Desirability function

**Figure 4:**
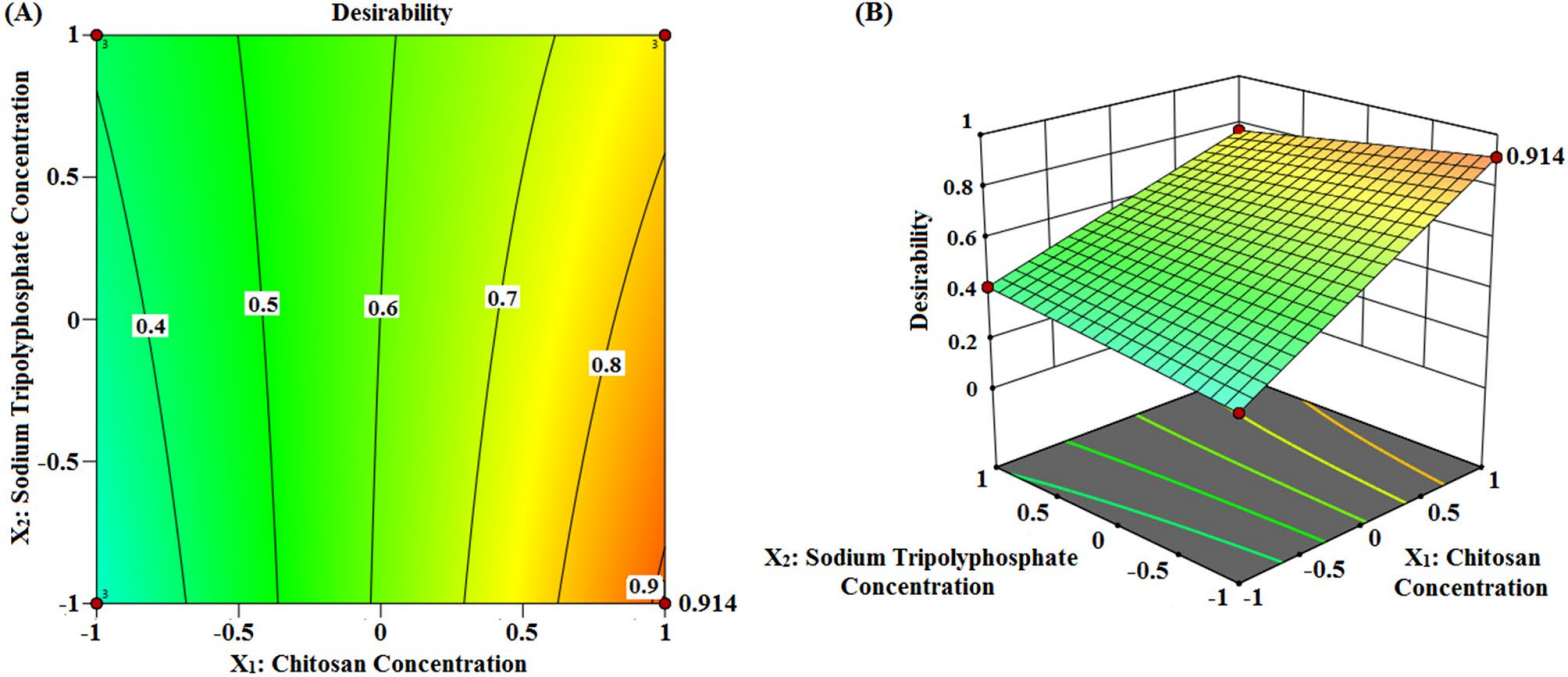
Optimization graph. (**A**) Contour and (**B**) three dimensional plots with desirability score using Design-Expert version 12 (/https://www.statease.com/software/design-expert).

Desirability function was utilized to identify the best formula out of 16 formulae. Accordingly, desirability was computed by the model to select the optimized formulation with reasonable particle size value along with the highest Q, ZP and high DEE %. The desirability score (Fig. 4) of the design was found to be 0.914, for F-9, which insinuated precise outcome of the design. As reported, the desirability values commonly ranged from 0 to 1. Values near to zero indicate imprecise outcomes of the design whereas those close to one imply precise ones^49^.

Therefore, this formula (F-9), with (X_1_ (+), X_2_ (−), X_3_ (+), X_4_ (+)), was considered as the optimized formula and subjected to additional elaborate investigations.

### Evaluation of formula 9 (F-9) of ALL-loaded CS/STPP NPs

#### Morphology

**Figure 5:**
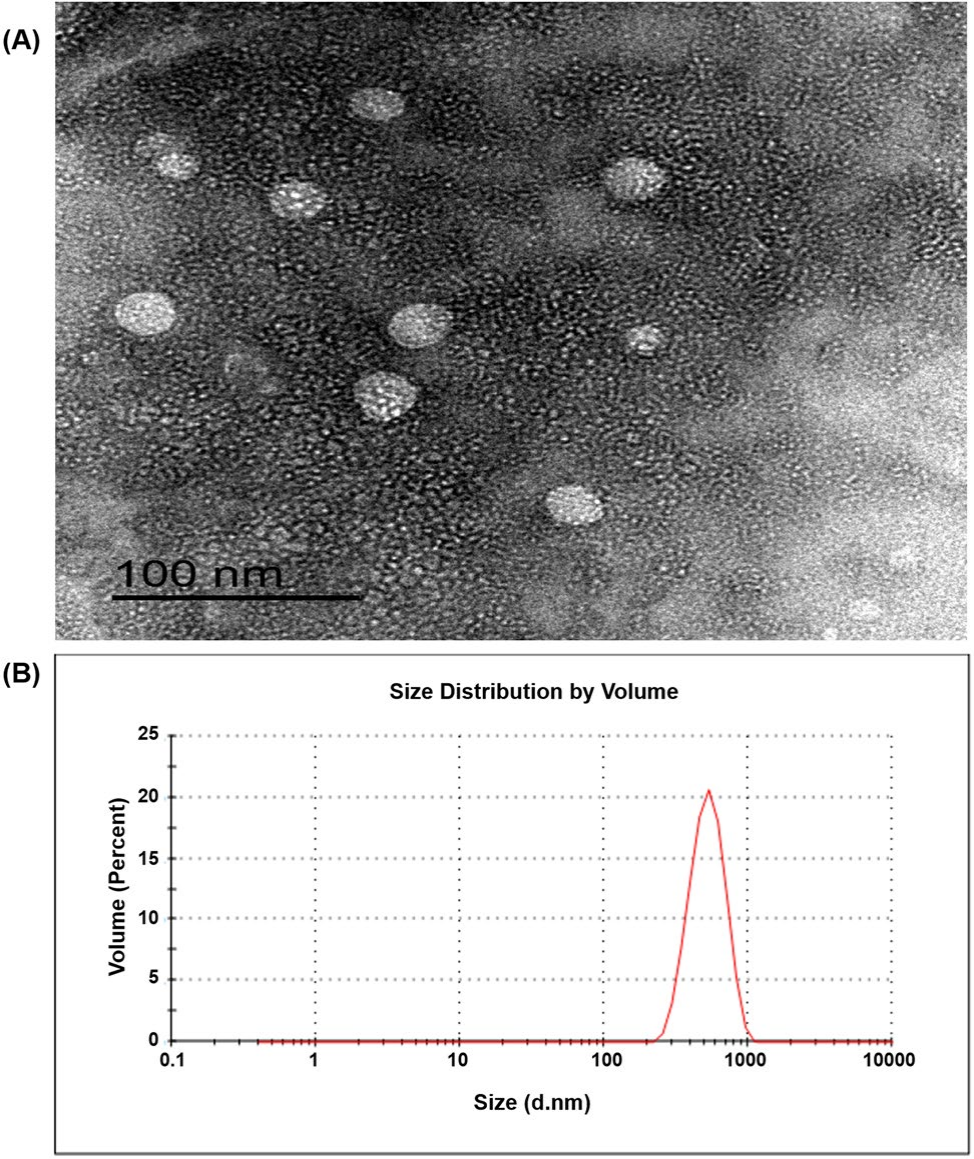
(**A**) TEM image of ALL-loaded CS/STPP NPs (F-9). The image was captured using Gatan Microscopy Suite Software, version 2.11.1404.0, (http://www.gatan.com/products/tem-analysis/gatan-microscopy-suite-software). (**B**) size distribution curve of ALL-loaded CS/STPP NPs (F-9).

TEM is a technique which uses an electron beam to image rather delicate nanoparticulate samples such as emulsions, vesicles, micelles, and NPs^17^. The photomicrographs of the TEM demonstrated nanostructures of spherical morphology (Fig. 5A). Notably, the particle size of the NPs estimated by TEM (< 200 nm) was diminished compared to that measured by Zetasizer (508.30 ± 13.35 nm, Fig. 5B), ascribed to the presence of the NPs in the dried state during TEM imaging, as previously reported^19^.

#### FT-IR

**Figure 6:**
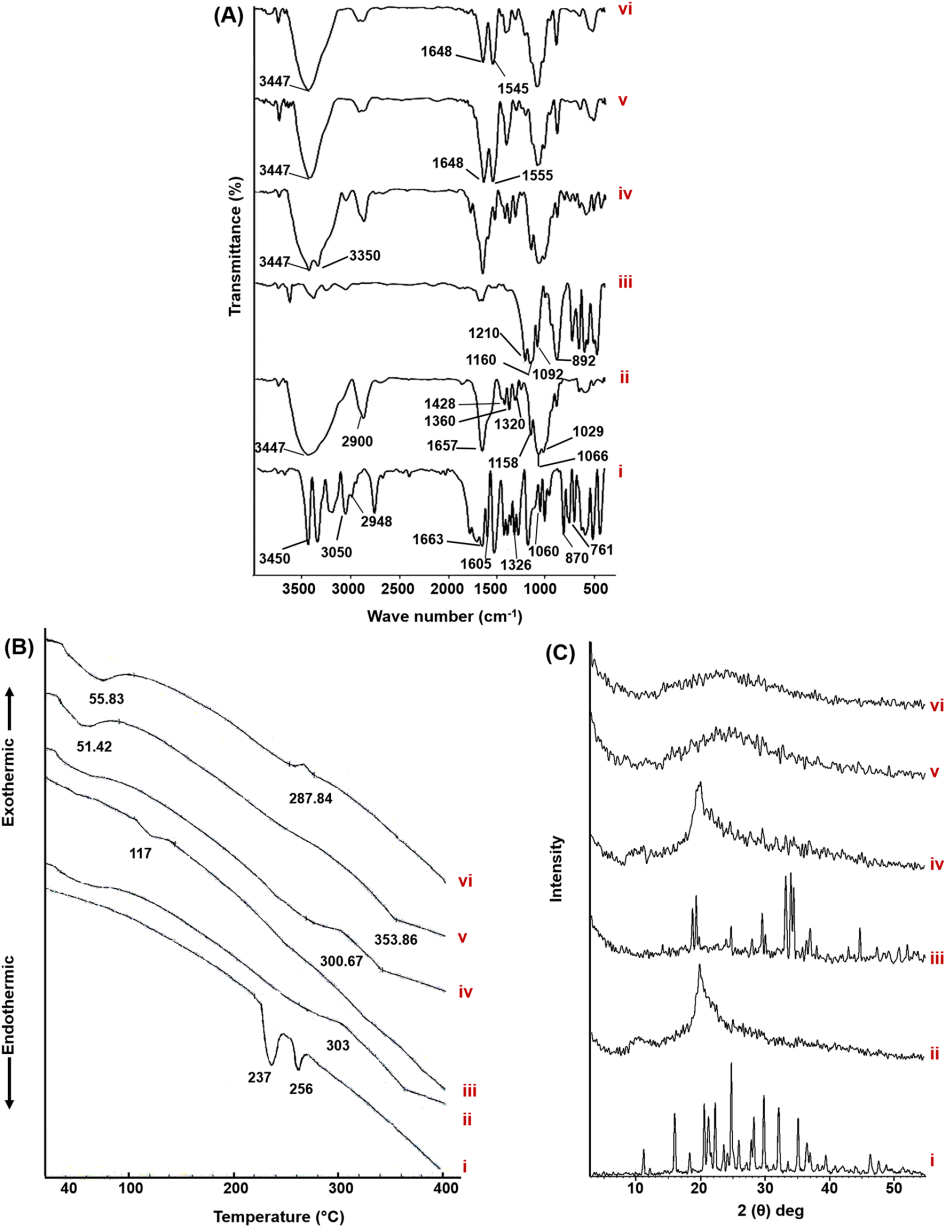
Solid characterization. (**A**) FT-IR spectra, (**B**) DSC thermograms, and (**C**) PXRD patterns of pure ALL (**i**), CS (**ii**), STPP (**iii**), physical mixture of ALL, CS and STPP (**iv**), plain CS/STPP NPs (**v**) and ALL-loaded CS/STPP NPs (F-9) (**vi**). DSC thermograms were fitted with a Shimadzu TA-60 series (https://www.ssi.shimadzu.com/products/thermal-analysis/labsolutions-ta-software.html).

As indicated in Fig. 6A, the FT-IR spectrum of ALL (i) shows distinct absorption bands between 3450 and 3050 cm^−1^ attributed to asymmetric and symmetric stretching vibrations of both –NH_2_ and –NH (imide as well as ring) groups. The peak at 2948 cm^−1^ is due to the stretching vibration of the aromatic C–H bond. Besides, the peaks at 1663 and 761 cm^−1^ are correlated to C=O (amide) stretching and C=O (ring) bending vibrations, respectively. The obvious bands appeared in the region of 1060–1605 cm^−1^ are due to the N–H in plane bending and rocking vibrations of NH_2_ group, while bands at 1326 and 870 cm^−1^ are attributed to C−N, of ring and ureidyl moiety, stretching vibrations^50,51^.

In the infrared spectrum of CS (ii), the distinctive peak located at 3447 cm^−1^ represents intermolecular hydrogen bonding and stretching vibration of –NH_2_ as well as –OH groups of CS. The peak at 2900 cm^−1^ typifies the stretching vibration of C–H from alkyl groups, whereas the presence of residual N-acetyl groups is confirmed by the peaks at around 1657 cm^−1^ (C=O stretching of amide I) and 1320 cm^−1^ (C−N stretching of amide III), respectively. Moreover, –CH_2_ bending besides –CH_3_ symmetrical deformations are confirmed by the presence of bands at 1428 and 1360 cm^−1^, respectively, while that at 1158 cm^−1^ can be attributed to asymmetric stretching of the C–O–C bridge. Besides, the bands at 1066 and 1029 cm^−1^ are related to C–O stretching. Analogous peaks were previously reported^52–54^.

The infrared shoulders at 1210, 1160, 1092 and 892 cm^−1^ appear indicating stretching vibration of P=O, symmetric and anti-symmetric stretching vibration of O–P=O, symmetric and anti-symmetric stretching vibration of the phosphate group (PO_3_) and stretching vibration of P–O–P bridge of STPP (iii), respectively^55,56^.

The spectrum of the physical mixture of F-9 (iv) namely; ALL (13 mg), CS (312.75 mg) and STPP (12.45 mg), shows the bands of CS, whereas those of both ALL and STPP are with diminished intensities as the outcome of dilution factor.

Plain and medicated CS/STPP NPs spectra were synchronized with each other, where C=O stretching of amide I peak of CS was shifted to 1648 cm^−1^ and a new peak appeared either at 1555 or 1545 cm^−1^, for plain (v) as well as medicated (vi) CS/STPP NPs spectra, respectively, (assigned to N–O–P stretching vibration) verifying the existence of ionic interactions between CS (amino groups) and STPP (phosphate groups) namely; cross-linking phenomenon.

Moreover, for medicated NPs, significant reduction at the intensity of the two aforementioned bands was observed. Such behavior might be attributed to ALL-CS interaction. These results correlate with earlier reports^55,57^.

Furthermore, ALL's distinctive peaks disappeared in the spectrum of medicated NPs, verifying its encapsulation in the NPs matrix besides the potential of interaction between ketonic (C=O) (ring) group of ALL with amino groups of CS of the NPs.

#### Thermal properties

Figure 6B illustrates the DSC thermograms of ALL, CS, STPP, and their physical mixture as well as plain and medicated CS/STPP (F-9).

Two characteristic endothermic peaks of ALL (i) were detected at 237 °C and 256 °C corresponding to its melting point besides its phase transformation from the solid crystalline phase to the gaseous phase, respectively^50^. CS thermogram (ii) disclosed an exothermic peak at 303 °C which indicates its degradation due to depolymerization and dehydration. STPP (iii) showed a weak endothermic peak at 117 °C, corresponding to its melting point, which is absent in the plain as well as medicated CS/STPP thermograms, presumably due to the ionic interaction with CS^58^.

Only one exothermic peak of CS with pronounced disappearance of those of ALL and STPP, as a consequence of dilution effect, was detected in the thermogram of their physical mixture (iv).

Interestingly, both plain (v) and medicated (vi) CS/STPP NPs thermograms experienced different endothermic and exothermic events, compared to pure CS and STPP, which might be accounted for the formation of a new structure with different thermal characteristics following cross-linking reactions^57^.

These thermal events were reached at lower temperature values in case of medicated rather than plain NPs, hence suggesting an interaction between ALL and CS. Such manner matches the FT-IR data. Moreover, the absence of ALL peaks was noticed in the thermogram of medicated NPs suggesting its entrapment in the matrix of the PNPs.

#### Powder x-ray diffraction (PXRD)

The PXRD patterns of ALL, CS, STPP, physical mixture, plain as well as ALL-loaded CS/STPP NPs (F-9) are illustrated in Fig. 6C. The PXRD pattern of ALL (i) displays intense diffraction peaks at angle 15.927°, 20.531°, 22.215°, 24.723°, 28.241°, 29.793°, 32.107°, and 35.122° (2θ), hence denoting the crystalline pattern of the drug.

On the contrary, the amorphous nature of CS (ii) was noticed^59^. STPP (iii) crystallinity was manifested by characteristic peaks at 2θ of 18.826, 19.397, 24.816, 29.664, 33.312, 34.118, and 34.555°^2^. The physical mixture (iv) shows the peaks of CS with clear absence of both ALL and STPP peaks due to their comparatively small amounts.

The diffractogram of the medicated ALL-loaded CS/STPP NPs (F-9) (vi) aligned with that of the plain one (v), besides absence of ALL's distinctive peaks. Therefore, entrapment of ALL within the CS matrix of the NPs in amorphous or molecular dispersed state exists. Previously, other loaded drugs in CS NPs showed a similar manner^60,61^.

#### In vitro ALL release study from ALL-loaded CS/STPP NPs (F-9)

**Figure 7:**
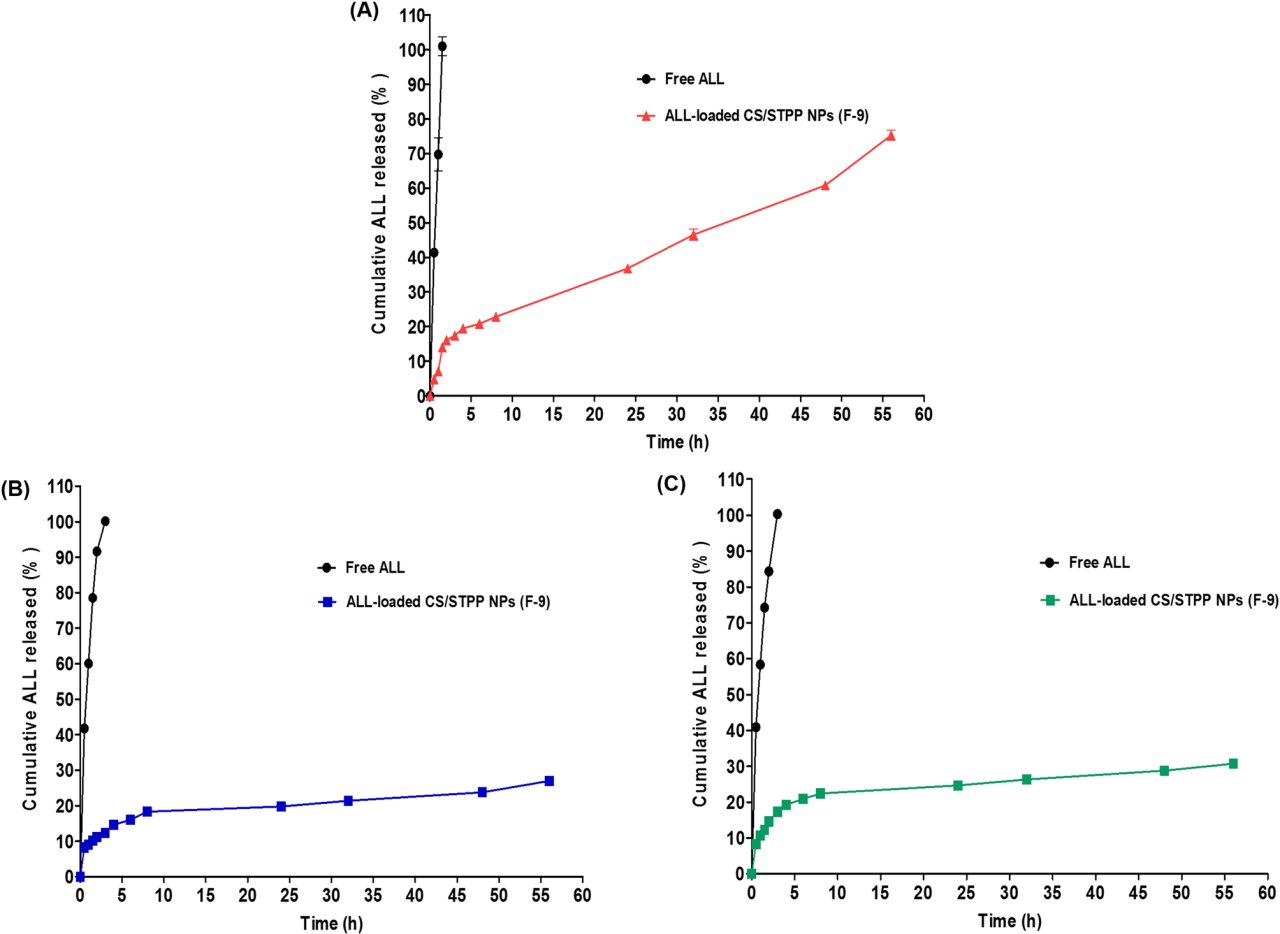
The in vitro release pattern of ALL from ALL-loaded CS/STPP NPs (F-9) in comparison with its diffusion from aqueous solution at three different pH values (**A**) pH 1.2, (**B**) pH 6.8 and (**C**) pH 7.4. Each point represents the mean ± SD (n = 3) and the graph was plotted using GraphPad Prism 5.00 (http://www.graphpad.com).

The in vitro release profiles of ALL from CS/STPP NPs (F-9) compared to its diffusion from aqueous solution were investigated using different simulated gastrointestinal fluids namely; solutions of 0.1 M HCl (pH 1.2 as simulated gastric fluid (SGF)), phosphate buffer (pH 6.8 as simulated intestinal fluid (SIF)) and phosphate buffer (pH 7.4 as simulated colonic fluid (SCF)) to mimic transit of orally administered formulations (Fig. 7).

Free ALL can be considered as an amphoteric molecule where it dissolved completely in all the release media, regardless the pH of the used media. ALL was completely diffused (100%) from its aqueous solution into the acidic pH of the stomach (pH 1.2) within one and half an hour (Fig. 7A). Similarly, 100% of the free ALL dissolved in 3 h at nearly neutral pH medium of the intestine (pH 6.8) as well as basic pH medium of the colon (pH 7.4) (Fig. 7B,C). Hydrogen-bond formation between C=O (amide) and NH_2_ groups of the drug and water (H_2_O) molecules of the release media may provoke the complete diffusion of free ALL.

On the contrary, ALL loaded in CS/STPP NPs exhibited a sustained release pattern at the different release media which is presumably due to ALL's slow diffusion from CS polymeric matrix. In pH 1.2, extensive CS swelling, as a consequence of the higher solubility of the polymer at lower pH, with subsequent erosion of NPs matrix may boost faster drug release (Fig. 7A). These results resembles an earlier study^62^. Additionally, besides the release characteristics of the formulation, the physicochemical properties of the drug might influence such behaviour. It could be speculated that the potential of interaction between ALL and CS, which is suggested via the obtained FT-IR as well as DSC data, might influence such sustained release behaviour. The prepared NPs could be considered as nanoscaffold or nanogel oral drug delivery systems.

#### Release kinetic

The kinetic release data of ALL from the optimized formula (F-9), in the SGF, was best fitted to Higuchi model (where diffusion-controlled drug release through the NPs matrix represents the rate determining step). While the Weibull model prevailed for entrapped ALL release from the NPs matrix from SIF as well as SCF, as depicted in Table 3.

**Table 3:** Kinetic analysis of the percentage drug released and that diffused from ALL-loaded CS/STPP NPs (F-9) and pure ALL, respectively (see Table 2 for F-9).

Formula	Coefficients of determination (R^2^)			Korsmeyer-Peppas			Weibull	
	Zero-order	First-order	Higuchi model	(R^2^)	Diffusional exponent (n)	Main transport mechanism	(R^2^)	β
F-9 (pH 1.2)	0.9539	0.9484	0.9670	0.9316	0.4473	Fickian	0.9464	0.5586
F-9 (pH 6.8)	0.7197	0.7585	0.8717	0.9604	0.2284	Fickian	0.9621	0.2495
F-9 (pH 7.4)	0.6453	0.6942	0.8271	0.9340	0.2238	Fickian	0.9418	0.2502
Free drug (pH 1.2)	0.9824	0.8250	0.9501	0.9354	0.4746	Fickian	–	–
Free drug (pH 6.8)	0.8665	0.8848	0.9888	0.9968	0.4820	Fickian	–	–
Free drug (pH 7.4)	0.8913	0.8055	0.9983	0.9990	0.4905	Fickian	–	–

Moreover, supplementary analysis by Korsmeyer-Peppas and Weibull mathematical models established a Fickian mechanism, n < 0.5 and β ≤ 0.75, elucidating that the drug release from NPs was substantially governed by diffusion. Similar kinetic behavior of other drugs loaded in CS/STPP NPs was previously reported^62,63^.

#### Mucoadhesive strength

The mucoadhesive characteristics of the optimized ALL-loaded CS/STPP NPs (F-9) was evaluated by measuring both the mucin-binding efficiency (%) and ZP on interaction with negatively-charged mucin. Such properties pave the way for prospective application as a drug delivery system.

#### Mucin-binding efficiency (%)

ALL-loaded CS/STPP NPs (F-9) possessed mucin-binding efficiency (%) value of 45.55 ± 3.76%. The mucoadhesive properties of CS are attributed to the electrostatic interaction between the amino groups of CS (positively-charged) and sialic acid residues of mucin (negatively-charged)^26^. As a consequence, the gastric residence time and cellular uptake of CS NPs, which are essential prerequisites for effective mucosal delivery of therapeutics, are increased^1, 2^.

#### ZP of CS/STPP NPs-mucin mixtures

**Figure 8:**
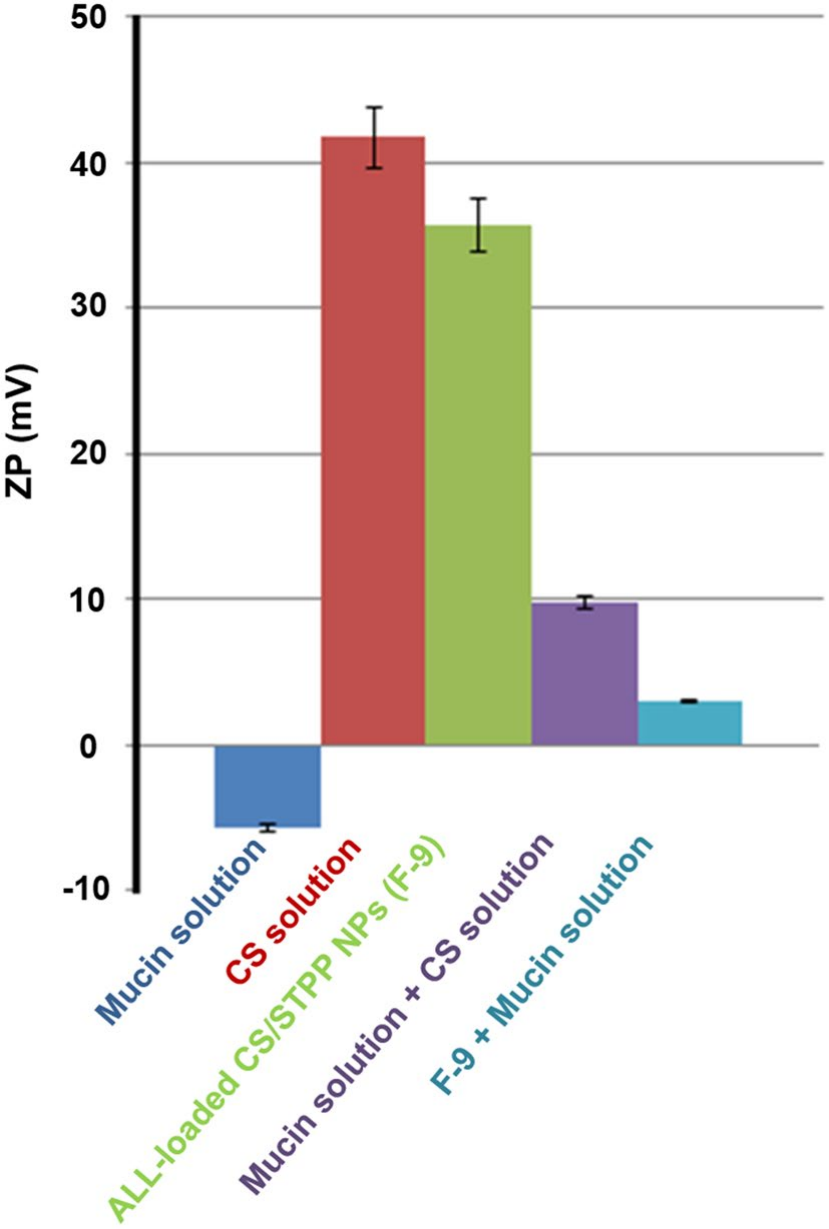
ζ-potential of mucin solution (0.5 mg/mL), CS solution (1.5% (w/v)), mucin solution + CS solution and ALL-loaded CS/STPP NPs (F-9) before and after incubation with mucin at 37 °C for 1 h. Results represents the mean ± SD (n = 3) and the graph was plotted using Microsoft Excel (2010), version 14.0.4734.1000, (www. Microsoft Office 2010.com).

In order to further emphasize the interactions between CS NPs and mucin, ZP was investigated (Fig. 8). ZP of mucin and CS solutions were − 5.67 ± 0.46 and + 41.73 ± 0.47 mV, respectively. It is well-known that positive charge of CS is due to the presence of ammonium ions (NH3^+^), however, negative charge of mucin is attributed to the ionization of sialic acid group (COO^−^). Incubation of ALL-loaded CS/STPP NPs (F-9) with mucin results in a significant decrease in ZP from 35.70 ± 0.82 to 3.02 ± 1.15 mV. Such reduction clearly reflected the strong electrostatic interaction between negatively-charged mucin and positively-charged CS^29^.

#### Short-term physical stability of ALL-loaded CS/STPP NPs (F-9)

In terms of stability, the effect of temperature (4 and 25 °C) on the physical stability of the optimized formula (F-9) was evaluated. However, owing to the storage of the samples into screw capped glass bottles, the influence of the relative humidity has not been considered^30^.

In the current study, ALL-loaded CS/STPP NPs (F-9) did not exhibit any physical changes in color and/or odor over a storage period of 3 months at (4 ± 1 °C) indicating its good stability. On the other hand, turbidity of the formulation was noticed at the end of storage at ambient conditions.

Table 4 outlines the values of particle size, PDI, ZP, and drug retention % of the optimized ALL-loaded CS/STPP NPs (F-9) stored at the two different storage temperatures. Using ANOVA for comparison with the freshly prepared ALL-loaded CS/STPP NPs samples (F-9), insignificant variation was detected concerning the evaluation parameters throughout the designated storage period at refrigerated condition. Otherwise, upon storage at room temperature, significant (*p* < 0.05) increase in particle size and PDI besides decrease in ZP were elucidated, while drug retention (%) was in the acceptable range (after 3 months). Such data emphasized the stability of F-9 at 4 ± 1 °C for 3 months demonstrated by uniform nanosize range together with homogenic dispersion. Similar findings, with regard to the storage stability study of CS/STPP NPs, were declared previously^30^.

**Table 4:** Particle size, PDI, ZP and drug retention % of ALL-loaded CS/STPP NPs aqueous dispersions (F-9) stored at refrigeration (4 ± 1 °C) and ambient conditions (see Table 2 for F-9).

Storage time	Evaluation parameters							
	Refrigeration conditions (4 ± 1 °C)				Ambient conditions			
	Particle size (nm)	PDI	ZP (mV)	Drug retention (%)	Particle size (nm)	PDI	ZP (mV)	Drug retention (%)
Zero time	508.30 ± 13.35	0.290 ± 0.02	+ 35.70 ± 0.82	100.00 ± 0.00	508.30 ± 13.35	0.290 ± 0.02	35.70 ± 0.82	100.00 ± 0.00
1 month	499.17 ± 0.93	0.282 ± 0.03	+ 33.60 ± 1.87	98.95 ± 5.29	501.57 ± 64.48	0.307 ± 0.02	+ 31.47 ± 0.99*	96.24 ± 3.62
3 months	504.60 ± 4.66	0.301 ± 0.02	+ 33.07 ± 1.11	98.202 ± 2.78	589.70 ± 8.97*^#^	0.509 ± 0.01*^#^	+ 14.80 ± 0.95*^#^	92.88 ± 3.33

Each value represents the mean ± SD (n = 3).

*Significant at *p* < 0.05 monthly vs. initial.

^#^Significant at *p* < 0.05 refrigeration vs. ambient conditions after 3 months.

#### Evaluation of AA against ethanol-induced gastric ulcer in rats

Ethanol-induced gastric ulcer in rats is a well-established pharmacological model for screening the AA of various drugs and natural products either as free or incorporated in pharmaceutical delivery systems. As documented, gastric ulcer caused by ethanol occurs via numerous mechanisms that include the trigger of mucosal oxidative stresses which leads to various dysregulation in NO generation, provoking inflammatory response causing release of inflammatory mediators such as IL-6 and TNF-α, that ultimately cause the destruction of the mucosal barrier^7,64^.

#### Macroscopic examination of gastric ulceration

**Figure 9:**
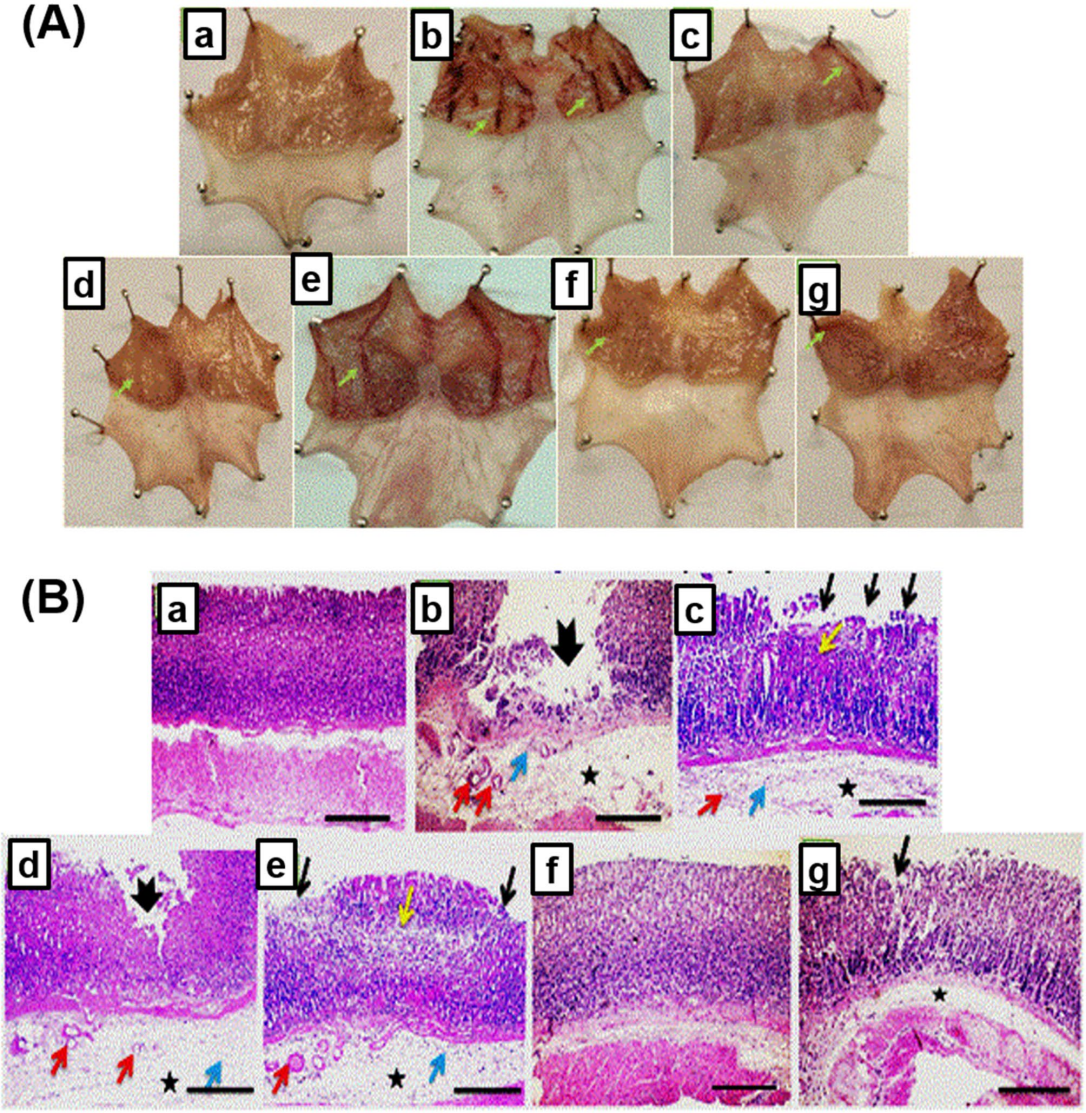
(**A**) Macroscopically investigation of mucosal lesions in glandular part of the stomachs. (a) N group, (b) ulcer group, (c) ome group, (d) plain group, (e) ALL group, (f) NanoAL group, and (g) NanoAH group. (**B**) Microscopical examination of glandular part of stomach sections stained by (H&E) (X: 100). (a) N group, (b) ulcer group, (c) ome group, (d) plain group, (e) ALL group, (f) NanoAL group, and (g) NanoAH group. Green arrow points to visual changes than the normal mucosa present in N group, thick black arrow points to mucosal ulceration, thin black arrow points to erosion, yellow arrow points to necrosis, asterisk points to edema, red arrow points to dilated blood vessel, and blue arrow points to leukocytic cells infiltration.

Figure 9A, taken personally by the author, represents the macroscopic gross appearance of freshly excised gastric mucosal tissues obtained from the different groups that were pretreated orally with either the free drug or the optimized CS/STPP NPs, along with the control groups. In addition, parameters for the assessment of the gastric injury were summarized in Table 5.

**Table 5:** Gastric AA evaluation parameters against ethanol-induced gastric injury in rats.

Group	UA (mm^2^)	UI	PI	pH
N	–	–	–	4.8 ± 0.05
Ulcer	165.2 ± 45.96	7.79 ± 2.71	–	3.4 ± 0.06^#^
Ome	27.33 ± 12.17***	1.59 ± 0.64	79.62	4.52 ± 0.07***
Plain	22.74 ± 14.45***	1.54 ± 0.79	80.18	3.8 ± 0.14*^,@^
ALL	23.16 ± 16.8***	1.45 ± 0.94	81.33	3.9 ± 0.02**^,@^
NanoAL	4.725 ± 3.55***	0.23 ± 0.13*	97.07	4.45 ± 0.11***^,^,%^
NanoAH	2.99 ± 1.32***	0.211 ± 0.11*	97.29	4.34 ± 0.07***^,^,%^

Data are expressed as mean ± SEM, ^#^*p* < 0.001 compared to N group, *p < 0.05 compared to ulcer group; **p < 0.01 compared to ulcer group; ***p < 0.001 compared to ulcer group; ^@^*p* < 0.001 compared to ulcer group; ^^^*p* < 0.01 compared to plain group; ^%^*p* < 0.05 compared to ALL group.

In the present study, ulcer group exhibited the most extensive damage of ethanol on the gastric lining when compared to the normal smooth lining found in the stomachs of N group (Fig. 9A-a,b). The finding is consistent with the previous studies indicating the reliability of ethanol as gastric ulcer inducer for the evaluation of the role of ALL in gastroprotection^7,9,64^.

As expected, the standard drug, Ome, (Fig. 9A-c) showed a visual reduction in ethanol-induced damage of the gastric lining as compared to the ulcer group. The carrier nano-particles itself, plain CS/STPP NPs (F-9), exhibited an improvement against ethanol when compared to the ulcer group and as comparative as the standard drug group, Ome, (Fig. 9A-d).

Administration of ALL-loaded CS/STPP NPs, in the doses 30 mg/kg (Fig. 9A-f) and 60 mg/kg (Fig. 9A-g), exhibited an almost normal lining with minimal observed damage as redness or signs of localized inflammation when compared to the free ALL with the high dose (60 mg/kg) (Fig. 9A-e) and with the ulcer group.

Parameters of gastric injury confirmed such findings (Table 5). Ulcer group exhibited the highest UA and UI besides the lowest pH values (165.2 ± 45.96 mm^2^, 7.79 ± 2.71 and 3.4 ± 0.06, respectively). Overproduction of HCl is an indication of the offensive circumstance resulted from the intake of ethanol. Ome group exhibited improvement in these mentioned parameters (UA = 27.33 ± 12.17 mm^2^, UI = 1.59 ± 0.64 and pH = 4.52 ± 0.07) inducing a PI of 79.6%. Interestingly, the plain group exhibited similar results, regarding the UA and AI, to Ome group invoking a PI of 80.18%.

Non-medicated CS-coated nanoparticles have been previously shown to have a great AA^65^. It was indicated that CS-based delivery systems support an enhancement in mucoadhesion ability through an interaction of the positively-charged CS-coated nanoparticles and the negatively-charged gastric mucosal content supporting a more potentiated AA for the systems^65^. Moreover, CS is known to exhibit acid-neutralizing capability by the gradual release of glucosamine residues into the gastric mucosa. Whereas, prevention of acid overproduction is well-known to accelerate ulcer healing.

Administration of ALL-loaded CS/STPP NPs, in the doses of 30 mg/kg (NanoAL group) and 60 mg/kg (NanoAH group), proved the most superiority as AA in enhancing the AA parameters; where the total UA was 4.725 ± 3.55 and 2.99 ± 1.32, respectively, and AI was 0.23 ± 0.13 and 0.211 ± 0.11, respectively, grossing a PI of 97% for both, compared to a PI of 81.33% for ALL group (60 mg/kg).

These findings proved not only the success of the CS/STPP carrier system as AA system by significantly reducing the damage of ethanol on the gastric mucosa compared to ulcer group, but also its ability to reach the same AA effect by reducing the medicated portion (ALL) to a half of the dose (from 60 to 30 mg/kg of ALL). The AA of ALL-loaded CS/STPP might be due to the inhibition of the gastric acid secretion as well by scoring pH values of 4.45 and 4.43, respectively. These findings would be further confirmed by histopathological, IHC, and biochemical studies.

### Histopathological examination

#### H&E stain

Histopathological imaging, using H&E stain, of the glandular portion of the stomach also added to such confirmations. For instance, normal glandular mucosa, submucosa and muscular layer were preserved in N group (Fig. 9B-a).

Meanwhile, sections of glandular stomachs from the ulcer group showed a deep ulceration reaching muscularis mucosa accompanied with extensive edema, dilated blood vessel and few leukocytic cells infiltrations in the underlying submucosal layer (Fig. 9B-b).

Pre-treatment with Ome or ALL (Fig. 9B-c and e) showed disrupted glandular pattern, mucosal necrosis and erosion with submucosal edema, dilated blood vessels and few leukocytic cells infiltrations, yet not as severe as in ulcer group. Similarly, sections of the glandular stomach from the plain group, treated with CS/STPP NPs, showed a superficial ulceration accompanied with extensive edema, dilated blood vessels and few leukocytic cells infiltrations in the underlying submucosal layer (Fig. 9B-d), also not as prominent as in the ulcer group.

Most interestingly, ALL-loaded CS/STPP NPs (F-9) exhibited the most noticeable protection against ethanol-induced gastric injury with almost normal appearance of the gastric sections showing milder focal mucosal erosion with very mild submucosal edema in both NanoAL and NanoAH groups (Fig. 9B-f and g). Noteworthy, the histopathological examination was in accordance with the PI of different rat groups supporting the potentiated AA of the medicated CS/STPP NPs.

#### Alcian blue stain

**Figure 10:**
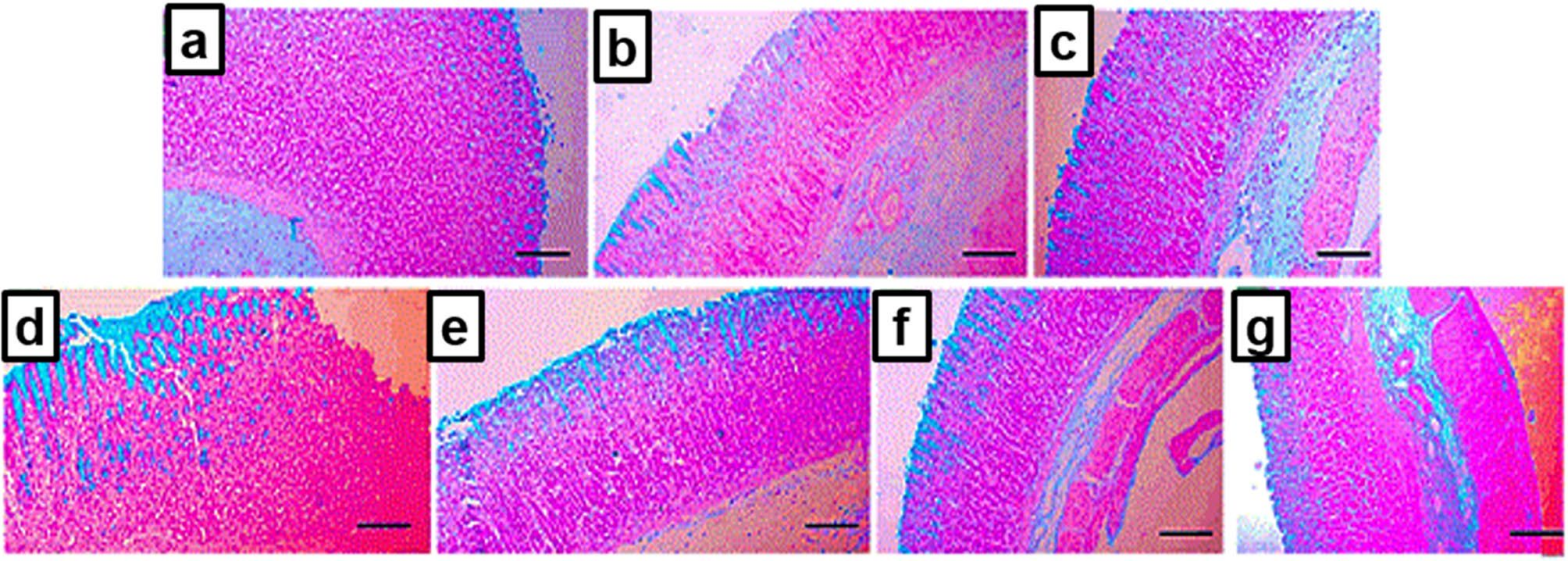
Histochemical staining of stomach sections of rats from the different treated groups with alcian blue stain to detect gastric mucus (X: 100). (**a**) N group, (**b**) ulcer group, (**c**) ome group, (**d**) plain group, (**e**) ALL group, (**f**) NanoAL group, and (**g**) NanoAH group.

Preserving the glycoprotein-based protecting layer of the gastric lumen is significantly important in prevention of the damaging effect of ethanol-induced gastric injury. Alcian blue-stained lining of the gastric lumen was discontinuous in the ulcer group (Fig. 10b) with signs of significant mucosal erosion and loss of basement membrane when compared to the normal and intact mucosa in N group (Fig. 10a).

Similarly, the plain group (Fig. 10d) exhibited an evident gastric mucosal erosion and lost basement membrane. Administration of ALL-sol reduced the damage to the gastric mucosa with minimal inflammation and erosions and allowing an intact basement membrane (Fig. 10e).

On the other hand, both Ome and NanoAH groups exhibited similar effects with healed gastric mucosa and no erosions (Fig. 10c,g). Even with decreasing the dose of ALL to be loaded on the CS/STPP NPs into (30 mg/kg), NanoAL group showed similar improvement to NanoAH and more superior to the carrier CS/STPP NPs alone, with minimal and almost normal gastric mucosa with no erosions or ulcerations (Fig. 10f).

As a matter of fact, mucus glycoproteins form a viscoelastic mucus gel layer protect and lubricate the underlying epithelium of gastric tissue, hence play a crucial defense role in the protection against gastric ulceration^2,66^.

The conspicuous effect of the medicated CS/STPP NPs to restore and preserve the mucus glycoproteins could be attributed to the benefits of both ALL and CS, firstly, the ability of orally administered ALL to induce gastric mucus production, via preserving the PGs "mainly prostaglandin E_2_ (PGE_2_)" content in case of ulceration, in addition to the viscous gel-forming mucoadhesive properties of CS^7,67^.

In the current study, such mucoadhesiveness was greatly confirmed by in vitro*-*in vivo correlation results (in vitro mucoadhesive strength study as shown in Fig. 8 and in vivo histochemical staining of the mucus glycoproteins).

#### Effect of ALL-loaded CS/STPP NPs (F-9) on the gastric oxidative stress

Oxidative stresses are critically involved in the pathogenesis of gastric ulceration. MDA is a lipid peroxidation marker which reflects the oxidative damage induced by reactive oxygen species (ROS) generated by ethanol. The overall result of ROS is the disruption of the GIT barrier, increasing its permeability and allowing the release of tissue-destroying inflammatory mediators, by the infiltrating neutrophils and macrophages, such as proteases and leukotrienes. Moreover, GSH is an antioxidant tripeptide present abundantly in cells, such as the gastric cells, to scavenge ROS and protect the cells against oxidative damage by regulating the redox status of the proteins in the mucosal cell-surface membrane.

**Figure 11:**
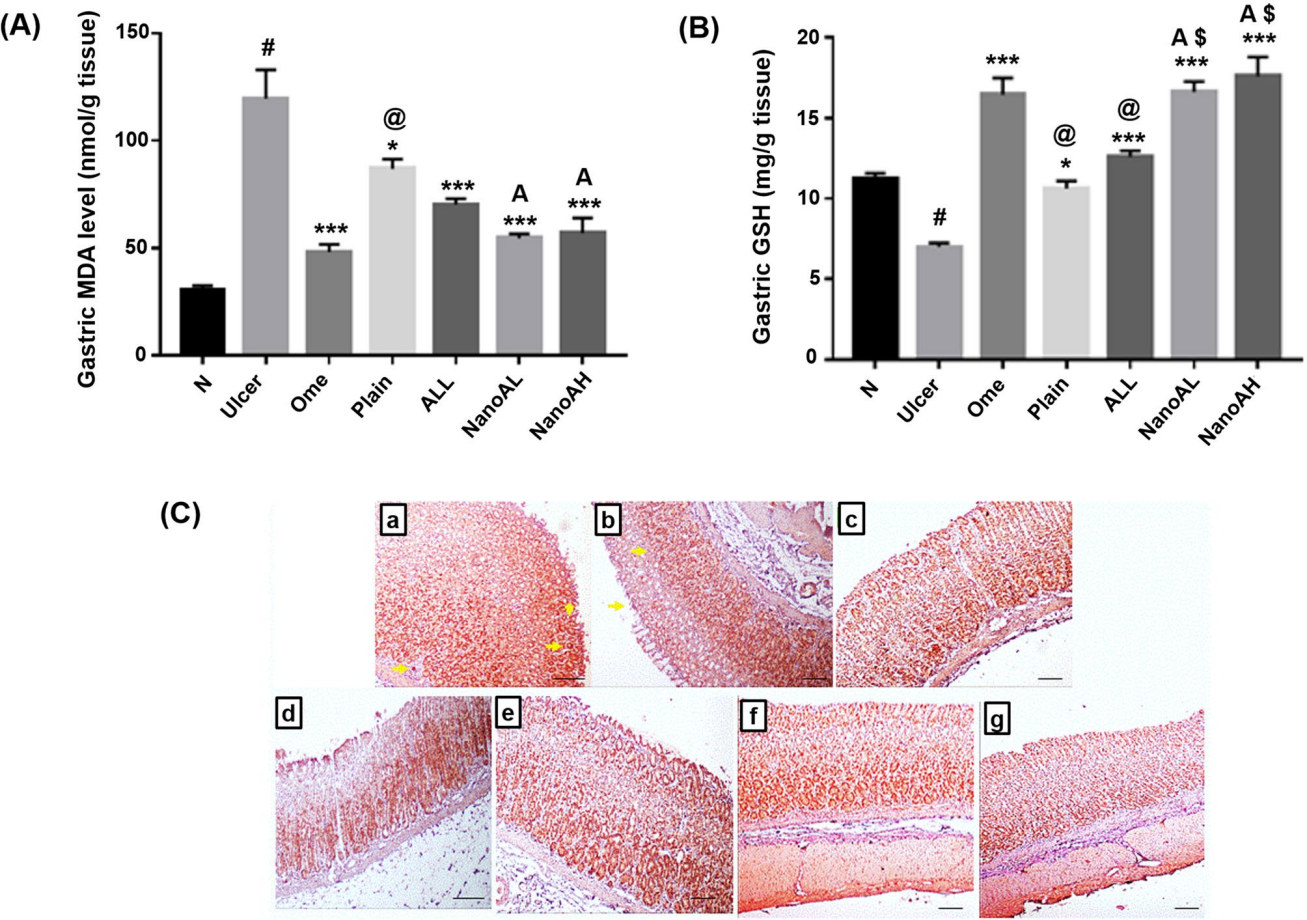
Oxidative status of (**A**) gastric MDA (nmol/g tissue) and (**B**) gastric GSH (mg/g tissue) in rats’ gastric tissues. (**C**) Immunostaining of Nrf-2 in rats’ gastric tissues (X: 100). (a) N group, (b) ulcer group, (c) ome group, (d) plain group, (e) ALL group, (f) NanoAL group, and (g) NanoAH group. X: 100. Data are expressed as mean ± SEM, ^#^*p* < 0.001 compared to N group, *p < 0.05 compared to ulcer group, ***p < 0.001 compared to ulcer group, ^@^*p* < 0.01 compared to ome group, ^$^*p* < 0.001 compared to ALL group, ^A^*p* < 0.05 compared to plain group using GraphPad Prism 5.00 (http://www.graphpad.com). Positive stainings are shown by yellow arrow.

Both MDA and GSH has been reported to be dysregulated by ethanol during the induction of gastric injury^68^. The gastric tissue levels of both MDA and GSH are shown in Fig. 11A,B.

The data demonstrated that the ulcer group exhibited the most significant oxidative damage induced by ethanol to the gastric tissues; expressed as a highly significant elevation of gastric MDA level (*p* ˂ 0.001), along with a depletion in gastric GSH level (*p* ˂ 0.001) in comparison with N group. Increased level of MDA in the ulcerated gastric mucosa has been reported to increase GSH consumption and a reliable indicator of the damage induced to the mucosal cell membrane^68^.

The standard drug, Ome, as expected by reducing the production of HCl and thus allowing the healing post ethanol-induced ulcer, was able to restore the oxidative balance by decreasing the gastric level of MDA and enhancing the gastric level of GSH.

Administration of CS/STPP NPs or the free ALL to the plain or ALL group, respectively, exhibited also a significant decrease in the gastric level of MDA and enhancement of gastric level of GSH as compared to ulcer group (*p* < 0.05 and *p* < 0.001, respectively) but not as Ome group. Both CS and ALL are well-documented for their antioxidant effects^7,68–70^.

The antioxidant effect of CS/STPP NPs is thought to be augmented by being loaded with ALL. Pre-treatment with ALL-loaded CS/STPP NPs (F-9), in both NanoAL and NanoAH groups, proved a more superiority to CS/STPP NPs alone, with significant reduction in the gastric levels of MDA, but elevation in GSH when compared to plain group alone (*p* < 0.05) and to the ulcer group (*p* < 0.001).

#### IHC detection of Nrf-2 expression

Nrf-2 is a transcription factor that regulates the expression of genes involved in different cellular functions, most importantly, the protection of the GIT against oxidative damage and, subsequently, inhibiting proinflammatory signaling^71^. Under normal condition, Nrf-2 acts by being translocated into the nucleus, where it binds to specific antioxidant response elements and upregulating the expression of certain antioxidant enzymes^72^.

In the ulcer group (Fig. 11C-b), IHC staining of Nrf-2 indicated a negative staining in the ulcerated mucosa, present in the area of the ethanol-induced gastric damage, with a positive staining in the normal gastric mucosa. In contrast, N group exhibited a strong positive staining through the gastric lining (Fig. 11C-a).

Tissue expression of Nrf-2 was found to be significantly reduced in ethanol-induced ulcer^71,73^. Moderate improvement in gastric Nrf-2 level was induced by both ALL and CS/STPP NPs (Fig. 11C-e and d), while the strongest staining was exhibited with groups that were administered either Ome or ALL-loaded CS/STPP NPs (30 mg/kg or 60 mg/kg) (Fig. 11C-c,f,g) when compared to the ulcer group (Table 6).

**Table 6:** Immunohistochemical score of gastric Nrf-2 and TNF-α.

Score	Nrf-2	TNF-α
N	+++	+
Ulcer	+	+++
Ome	+++	++
Plain	++	+++
ALL	++	++
NanoAL	+++	++
NanoAH	+++	++

(+) weak staining, (++) moderate staining, and (+++) strong staining.

The effect of ALL on Nrf-2 may be explained through its antioxidant activity. Several phytochemical compounds have been shown to upregulate a positive effect on Nrf-2, exerting their antioxidant activity, most specifically in ethanol-induced ulcer^74^. In addition, Nrf-2 activation has been proven to be one of the antioxidant mechanisms possessed by CS^75^. Combining both CS and ALL as ALL-loaded CS/STPP NPs (F-9) augmented its ability to induce Nrf-2 and allowing Nrf-2-induced antioxidant-response against ethanol-induced gastric injury.

#### Effect of ALL-loaded CS/STPP NPs (F-9) on serum and gastric pro-inflammatory mediators

Inflammation plays a significant role in stimulating gastric tissue infiltration with neutrophils, macrophages and other lymphocytes inducing toxic metabolites, ROS generations and lysosomal enzymes promoting a local tissue damage associated with ulcer induction^76^. IL-6 is a key regulatory cytokine in stimulating and orchestrating the actions of these inflammatory cells^77^.

**Figure 12:**
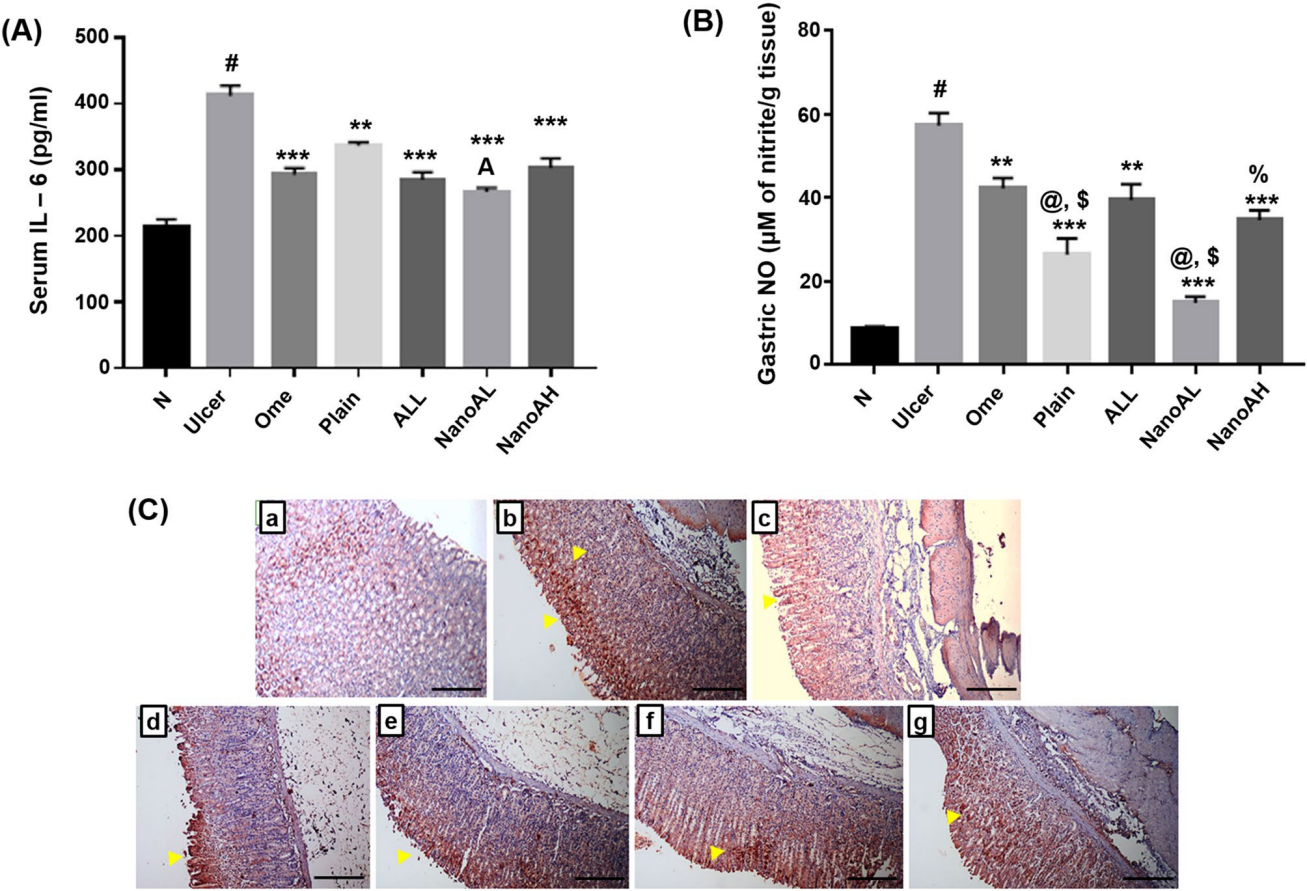
Levels of pro-inflammatory mediators; (**A**) serum level of IL-6 (pg/ml) and (**B**) gastric level of NO (μM of nitrite/g tissue). (**C**) Immunostaining of TNF-α in rats’ gastric tissues (X: 100). (a) N group, (b) ulcer group, (c) ome group, (d) plain group, (e) ALL group, (f) NanoAL group, and (g) NanoAH group. X: 100. Data are expressed as mean ± SEM, ^#^*p* < 0.001 compared to N group, **p < 0.01 compared to ulcer group ***p < 0.001 compared to ulcer group, ^@^*p* < 0.01 compared to Ome group, ^$^*p* < 0.001 compared to ALL group, ^A^*p* < 0.01 compared to plain group, ^%^*p* < 0.001 compared to NanoAL group using GraphPad Prism 5.00 (http://www.graphpad.com). Positive stainings are shown by yellow arrow.

Serum level of IL-6 was significantly elevated in the ulcer group (*p* < 0.001) when compared to N group (Fig. 12A). Previous studies have shown significant elevation of IL-6 in both serum and gastric tissues during ulcer^76,78^.

Administration of the nanocarrier, CS/STPP NPs (F-9), to the plain group significantly reduced the serum level of IL-6 (*p* < 0.01) when compared to the ulcer group. Interestingly, all treatment groups with the standard drug (Ome) or ALL or the ALL-loaded CS/STPP NPs exhibited similar significance as anti-inflammatory agents against the ethanol-induced ulcer (*p* < 0.001). Previously, the association between the activation of Nrf-2 and the prevention of certain pro-inflammatory cytokines, including IL-6 and IL-1β has been reported^79^.

Gastric NO has been proven to be sometimes contradictory^80^. In our study, ulcer group exhibited a significant elevation in the gastric level of NO when compared to N group (Fig. 12B). Ethanol-induced gastric lesions are well associated with the elevation of the gastric NO^81^.

Although, NO has been reported to act as an anti-inflammatory inducer, allowing a better vascular support and mucus production. However, when excessively produced, NO could induce ROS and the subsequent promotion of inflammation. These contradictions depend on the expression and level of the enzyme nitric oxide synthase (NOS) which is majorly expressed in three isoforms; neuronal (nNOS), endothelial (eNOS) and inducible (iNOS). The latter is well-known to be expressed under pathological circumstances involving the exposure to cytokines. Overactivity of iNOS causes excessive production of NO contributing to the gastric mucosal injury and dysfunction^82^. Therefore, the inhibition of NO overproduction is regarded as a preferable tactic for the therapeutic intervention in inflammatory diseases.

Group administered with plain CS/STPP NPs (F-9) exhibited a significant reduction in the gastric level of NO when compared to ulcer, ALL (*p* < 0.001) and Ome (*p* < 0.01) groups. In a previous study, a modified form of CS was found to significantly reduce lipopolysaccharide induced-iNOS and subsequently NO in macrophage cells^83^.

Moreover, groups pre-treated with ALL-loaded CS/STPP NPs as (30 or 60 mg/kg), NanoAL and NanoAH groups, exhibited a significant reduction of the gastric NO when compared to the ulcer group (*p* < 0.001). Interestingly, NanoAL exhibited the most significant reduction in the gastric level of NO as compared to ALL-sol (*p* < 0.001) and Ome (*p* < 0.01). Additionally, the aforementioned group showed a decrease in the gastric level of NO when compared to NanoAH group (*p* < 0.001).

The pro-inflammatory marker, TNF-α, was detected by immunostaining in the gastric sections indicating a strong positive reaction in ulcer group (Fig. 12C-b) at the gastric glands at the base and margin of the ulcerated mucosa compared to almost negative staining in N group (Fig. 12C-a).

The increased level of TNF-α by ethanol, during ulcer induction, is well documented^7,84^. TNF-α is the orchestrating molecule of several pathological pathways that could even be deemed contrasting in their action. For instance, it derives the extrinsic aspect of the cellular apoptosis, as well as, the upregulation of the nuclear factor kappa-light-chain-enhancer of activated B cells (NF-κB), which further enhances the transcription of other pro-inflammatory cytokines, such as Interleukin-1-beta (IL1β) and TNF-α^85^. Furthermore, TNF-α prevents gastric microcirculation around the ulcerated mucosa and thus delays its healing^86^. Interestingly, dysregulation of TNF-α plays significantly in the development of gastric cancer^87^.

In our study, Ome group exhibited a moderate positive cytoplasmic reaction, specifically, in the healed gastric glands (Fig. 12C-c), indicating an expected protecting effect of the drug against ethanol-induced gastric ulcer. While, plain CS/STPP NPs exhibited a mild reduction in the gastric TNF-α, specifically, at the ulcerated area but strong positive cytoplasmic reaction in the glands at the base of the ulcer (Fig. 12C-d). Administration of ALL-sol showed moderate positive cytoplasmic reaction of TNF-α (Fig. 12C-e). While, administration of ALL-loaded CS/STPP NPs (F-9), to either NanoAL or NanoAH group (Fig. 12C-f and g), reduced the level of TNF-α to a near normal pattern, with moderate focal positive cytoplasmic reaction in the healed gastric glands (Table 6).

The effect of the drug on reducing the level of TNF-α localization is probably due to ALL's ability to preserve PGE_2_ content in gastric mucosa, since the synthesis of TNF-α is inhibited by PGs^7,88^. The effect is also could be attributed to the design of nanoparticles allowing ALL to be sustainably released at the site of gastric ulcer affecting the upregulation of TNF-α and preventing its role in gastric injury and stripping of the mucin protective layer.

## Conclusion

Concisely, ALL-loaded CS/STPP NPs were successfully prepared by ionotropic gelation technique. A full 2^4^ factorial design paradigm was employed to optimize the DMPs of the prepared NPs for further characterization and extensive investigation of the optimized formula. FT-IR and DSC results, of the optimized formula, confirmed ALL entrapment in the matrix of the PNPs system. TEM imaging displays nanosized structures with spherical morphology. The optimized formula (F-9) was physically stable at the tested temperatures. The conspicuous in vivo AA of ALL-loaded CS/STPP NPs was asserted by histopathological, IHC, and biochemical studies. These auspicious preclinical data deserve profound attention for prospective clinical evaluation of the developed phyto-pharmaceutical polymeric nanoparticulate system as effective and safe natural anti-ulcer therapy instead of the available ones that exhibited numerous drawbacks.

## Supplementary Information

Supplementary Table S1.

Supplementary Table S2.

## Data Availability

All raw and analyzed data as well as the materials are available in this study.

## References

[1] Dudhani AR, Kosaraju SL (2010). Bioadhesive chitosan nanoparticles: Preparation and characterization. Carbohydr. Polym..

[2] Anter HM, Abu Hashim II, Awadin W, Meshali MM (2019). Novel chitosan oligosaccharide-based nanoparticles for gastric mucosal administration of the phytochemical “apocynin”. Int. J. Nanomedicine.

[3] Grenha A (2012). Chitosan nanoparticles: A survey of preparation methods. J. Drug Target..

[4] Iswanti FC (2019). Preparation, characterization, and evaluation of chitosan-based nanoparticles as CpG ODN carriers. Biotechnol. Biotechnol. Equip..

[5] Al Asmari A (2015). Vanillin abrogates ethanol induced gastric injury in rats via modulation of gastric secretion, oxidative stress and inflammation. Toxicol. Rep..

[6] Al-Nimer MSM, Wahbee Z (2017). Ultraviolet light assisted extraction of flavonoids and allantoin from aqueous and alcoholic extracts of *Symphytum officinale*. J. Intercult. Ethnopharmacol..

[7] da Silva DM (2018). Effect of allantoin on experimentally induced gastric ulcers: Pathways of gastroprotection. Eur. J. Pharmacol..

[8] Selamoglu Z, Dusguna C, Akgulb H, Gulhanc MF (2017). In-vitro antioxidant activities of the ethanolic extracts of some contained-allantoin plants. Iran J. Pharm. Res..

[9] Eslami-farsani M, Moslehi A, Hatami-shahmir A (2018). Allantoin improves histopathological evaluations in a rat model of gastritis. Physiol. Int..

[10] Lopez-Lopez J (2015). Efficacy of chlorhexidine, dexpanthenol, allantoin and chitosan gel in comparison with bicarbonate oral rinse in controlling post-interventional inflammation, pain and cicatrization in subjects undergoing dental surgery. Curr. Med. Res. Opin..

[11] Madrazo-Jiménez M (2016). The effects of a topical gel containing chitosan, 0,2% chlorhexidine, allantoin and despanthenol on the wound healing process subsequent to impacted lower third molar extraction. Med. Oral Patol. Oral Cir. Bucal..

[12] Fedosov PA (2017). Preclinical study of the efficacy and safety of wound healing gel containing chitosan, taurine and allantoin. Res. Result Pharmacol. Clin. Pharmacol..

[13] Svetlichny G (2015). Solid lipid nanoparticles containing copaiba oil and allantoin: Development and role of nanoencapsulation on the antifungal activity. Pharmazie..

[14] Ke M (2016). Allantoin-loaded porous silica nanoparticles/polycaprolactone nanofiber composites: Fabrication, characterization, and drug release properties. RSC Adv..

[15] Manca ML (2016). Combination of argan oil and phospholipids for the development of an effective liposome-like formulation able to improve skin hydration and allantoin dermal delivery. Int. J. Pharm..

[16] Menezes JESA (2020). Preparation, structural and spectroscopic characterization of chitosan membranes containing allantoin. J. Mol. Struct..

[17] Aman RM, Abu Hashim II, Meshali MM (2018). Novel chitosan-based solid-lipid nanoparticles to enhance the bio-residence of the miraculous phytochemical “apocynin”. Eur. J. Pharm. Sci..

[18] Calvo P, Remuñan-López C, Vila-Jato JL, Alonso MJ (1997). Chitosan and chitosan/ethylene oxide-propylene oxide block copolymer nanoparticles as novel carriers for proteins and vaccines. Pharm. Res..

[19] Hashad RA, Ishak RA, Fahmy S, Mansour S, Geneidi AS (2016). Chitosan-tripolyphosphate nanoparticles: Optimization of formulation parameters for improving process yield at a novel pH using artificial neural networks. Int. J. Biol. Macromol..

[20] Shah M, Pathak K (2010). Development and statistical optimization of solid lipid nanoparticles of simvastatin by using 2^3^ full-factorial design. AAPS PharmSciTech..

[21] Higuchi T (1963). Mechanism of sustained action medication: Theoretical analysis of rate of release of solid drugs dispersed in solid matrix. J. Pharm. Sci..

[22] Korsmeyer RW, Gurny R, Doelker E, Buri P, Peppas NA (1983). Mechanism of solute release from porous hydrophilic polymers. Int. J. Pharm..

[23] Azadi S, Ashrafi H, Azadi A (2017). Mathematical modeling of drug release from swellable polymeric nanoparticles. J. APP Pharm. Sci..

[24] Lorenzo-Lamosa ML, Remuñán-López C, Vila-Jato JL, Alonso MJ (1998). Design of microencapsulated chitosan microspheres for colonic drug delivery. J. Control Release..

[25] Chuah LH, Billa N, Roberts CJ, Burley JC, Manickam S (2013). Curcumin-containing chitosan nanoparticles as a potential mucoadhesive delivery system to the colon. Pharm. Dev. Technol..

[26] Dyawanapelly S, Koli U, Dharamdasani V, Jain R, Dandekar P (2016). Improved mucoadhesion and cell uptake of chitosan and chitosan oligosaccharide surface-modified polymer nanoparticles for mucosal delivery of proteins. Drug Deliv. Transl. Res..

[27] De Campos AM, Diebold Y, Carvalho ELS, Sánchez A, Alonso MJ (2004). Chitosan nanoparticles as new ocular drug delivery systems: In vitro stability, in vivo fate, and cellular toxicity. Pharm. Res..

[28] Fathalla ZM, Khaled KA, Hussein AK, Alany RG, Vangala A (2016). Formulation and corneal permeation of ketorolac tromethamine-loaded chitosan nanoparticles. Drug Dev. Ind. Pharm..

[29] Hejjaji EM, Smith AM, Morris GA (2018). Evaluation of the mucoadhesive properties of chitosan nanoparticles prepared using different chitosan to tripolyphosphate (CS:TPP) ratios. Int. J. Biol. Macromol..

[30] Altaani BM, Suhair SA, Haddad RH, Abu-Dahab R (2019). Preparation and characterization of an oral norethindrone sustained release/controlled release nanoparticles formulation based on chitosan. AAPS PharmSciTech..

[31] El-Naga RN (2015). Apocynin protects against ethanol-induced gastric ulcer in rats by attenuating the upregulation of NADPH oxidases 1 and 4. Chem. Biol. Interact..

[32] Florentino FI (2016). Antinociceptive and anti-inflammatory effects of memora nodosa and allantoin in mice. J. Ethnopharmacol..

[33] Raish M (2018). Momordica charantia polysaccharides ameliorate oxidative stress, inflammation, and apoptosis in ethanol-induced gastritis in mucosa through NF-kB signaling pathway inhibition. Int. J. Biol. Macromol..

[34] Ibrahim MY (2016). Acute toxicity and gastroprotection studies of a new schiff base derived manganese (II) complex against HCl/ethanol-induced gastric ulcerations in rats. Sci. Rep..

[35] Bai K, Hong B, Tan R, He J, Hong Z (2020). Selenium nanoparticles-embedded chitosan microspheres and their effects upon alcohol-induced gastric mucosal injury in rats: Rapid preparation, oral delivery, and gastroprotective potential of selenium nanoparticles. Int. J. Nanomedicine..

[36] Zaghloul R (2017). Hepatoprotective effect of hesperidin in hepatocellular carcinoma: Involvement of Wnt signaling pathways. Life Sci..

[37] Eisa N, ElSherbiny N, Shebl A, Eissa L, El-Shishtawy M (2015). Phenethyl isothiocyanate potentiates anti-tumour effect of doxorubicin through Akt-dependent pathway. Cell Biochem. Funct..

[38] Asasutjarit R (2007). Effect of solid lipid nanoparticles formulation compositions on their size, zeta potential and potential for in vitro pHIS-HIV-Hugag transfection. Pharm. Res..

[39] Mohammed MA, Syeda JT, Wasan KM, Wasan EK (2017). An overview of chitosan nanoparticles and its application in non-parenteral drug delivery. Pharmaceutics..

[40] Khattab A, Zaki N (2017). Optimization and evaluation of gastroretentive ranitidine HCl microspheres by using factorial design with improved bioavailability and mucosal integrity in ulcer model. AAPS PharmSciTech..

[41] Deng Q, Zhou C, Luo B (2006). Preparation and characterization of chitosan nanoparticles containing lysozyme. Pharm. Biol..

[42] Katas, H., Hussain, Z. & Ling, T. C. Chitosan nanoparticles as a percutaneous drug delivery system for hydrocortisone. *J. Nanomater.* **2012**, ID 372725 (2012).

[43] Zhang H (2016). Chitosan-based nanoparticles for improved anticancer efficacy and bioavailability of mifepristone. Beilstein J. Nanotechnol..

[44] Puhl AC (2011). Preparation and characterization of polymeric nanoparticles loaded with the flavonoid luteolin, by using factorial design. Int. J. Drug Deliv..

[45] Servat-Medina L (2015). Chitosan–tripolyphosphate nanoparticles as *Arrabidaea chica* standardized extract carrier: Synthesis, characterization, biocompatibility, and antiulcerogenic activity. Int. J. Nanomedicine..

[46] Chiesa E (2019). Staggered herringbone microfluid device for the manufacturing of chitosan/TPP nanoparticles: Systematic optimization and preliminary biological evaluation. Int. J. Mol. Sci..

[47] Tzeyung AS (2019). Fabrication, optimization, and evaluation of rotigotine-loaded chitosan nanoparticles for nose-to-brain delivery. Pharmaceutics..

[48] Honary S, Zahir F (2013). Effect of zeta potential on the properties of nano-drug delivery systems—A review (part 2). Trop. J. Pharm. Res..

[49] Kumar L, Reddy MS, Managuli RS, Pai KG (2015). Full factorial design for optimization, development and validation of HPLC method to determine valsartan in nanoparticles. Saudi Pharm. J..

[50] Kuş N, Bayarı SH, Fausto R (2009). Thermal decomposition of allantoin as probed by matrix isolation FTIR spectroscopy. Tetrahedron.

[51] Alam MJ, Ahmad S (2015). FTIR, FT-Raman, UV–visible spectra and quantum chemical calculations of allantoin molecule and its hydrogen bonded dimers. Spectrochim. Acta A Mol. Biomol. Spectrosc..

[52] Othayoth R, Mathi P, Bheemanapally K, Kakarla L, Botlagunta M (2015). Characterization of vitamin–cisplatin-loaded chitosan nano-particles for chemoprevention and cancer fatigue. J. Microencapsul..

[53] Queiroz MF, Melo KRT, Sabry DA, Sassaki GL, Rocha HAO (2014). Does the use of chitosan contribute to oxalate kidney stone formation?. Mar. Drugs..

[54] Ahmed R (2018). Novel electrospun chitosan/polyvinyl alcohol/zinc oxide nanofibrous mats with antibacterial and antioxidant properties for diabetic wound healing. Int. J. Biol. Macromol..

[55] Loutfy SA (2016). Synthesis, characterization and cytotoxic evaluation of chitosan nanoparticles: In vitro liver cancer model. Adv. Nat. Sci. Nanosci. Nanotechnol..

[56] Tomaz AF (2018). Ionically crosslinked chitosan membranes used as drug carriers for cancer therapy application. Materials..

[57] Azevedo JR (2011). Physical and chemical characterization insulin-loaded chitosan-TPP nanoparticles. J. Therm. Anal. Calorim..

[58] Nair RS, Morris A, Billa N, Leong C-O (2019). An evaluation of curcumin-encapsulated chitosan nanoparticles for transdermal delivery. AAPS PharmSciTech..

[59] de Queiroz Antonino RSCM (2017). Preparation and characterization of chitosan obtained from shells of shrimp (*Litopenaeus vannamei* Boone). Mar. Drugs..

[60] Papadimitriou S, Bikiaris D, Avgoustakis K, Karavas E, Georgarakis M (2008). Chitosan nanoparticles loaded with dorzolamide and pramipexole. Carbohydr. Polym..

[61] Harisa GI, Badran MM, Attia SM, Alanazi FK, Shazly GA (2018). Influence of pravastatin chitosan nanoparticles on erythrocytes cholesterol and redox homeostasis: An in vitro study. Arab J. Chem..

[62] Kulpreechanan N, Sorasitthiyanukarn FN (2020). Evaluation of in vitro release kinetics of capsaicin-loaded chitosan nanoparticles using DDSolver. Int. J. Res. Pharm. Sci..

[63] Alqahtani FY (2019). Preparation, characterization, and antibacterial activity of diclofenac-loaded chitosan nanoparticles. Saudi Pharm. J..

[64] Zhou D (2020). Gastroprotective effect of gallic acid against ethanol-induced gastric ulcer in rats: Involvement of the Nrf2/HO-1 signaling and anti-apoptosis role. Biomed. Pharmacother..

[65] Abd El Hady WE, Mohamed EA, Soliman OA, El-Sabbagh HM (2019). In vitro-in vivo evaluation of chitosan-PLGA nanoparticles for potentiated gastric retention and anti-ulcer activity of diosmin. Int. J. Nanomedicine..

[66] Prathima S, Harendra Kumar ML (2012). Mucin profile of upper gastrointestinal tract lesions. J. Clin. Biomed. Sci..

[67] Ito M, Ban A, Ishihara M (2000). Anti-ulcer effects of chitin and chitosan, healthy foods, in rats. Jpn. J. Pharmacol..

[68] Anandan R, Nair PG, Mathew S (2004). Anti-ulcerogenic effect of chitin and chitosan on mucosal antioxidant defence system in HCl-ethanol-induced ulcer in rats. J. Pharm. Pharmacol..

[69] Sousa F, Guebitz GM, Kokola V (2009). Antimicrobial and antioxidant properties of chitosan enzymatically functionalized with flavonoids. Process Biochem..

[70] Avelelas F (2019). Antifungal and antioxidant properties of chitosan polymers obtained from nontraditional *Polybius henslowii* sources. Mar. Drugs..

[71] Badr AM, El-Orabi NF, Ali RA (2019). The implication of the crosstalk of Nrf2 with NOXs, and HMGB1 in ethanol-induced gastric ulcer: Potential protective effect is afforded by raspberry ketone. PLoS ONE.

[72] Pérez S, Taléns-Visconti R, Rius-Pérez S, Finamor I, Sastre J (2017). Redox signaling in the gastrointestinal tract. Free Radic. Biol. Med..

[73] Yanaka A (2018). Role of NRF2 in protection of the gastrointestinal tract against oxidative stress. J. Clin. Biochem. Nutr..

[74] Zhao N, Guo FF, Xie KQ, Zeng T (2018). Targeting Nrf-2 is a promising intervention approach for the prevention of ethanol-induced liver disease. Cell Mol. Life Sci..

[75] Khodagholi F, Eftekharzadeh B, Maghsoudi N, Rezaei PF (2010). Chitosan prevents oxidative stress-induced amyloid beta formation and cytotoxicity in NT2 neurons: Involvement of transcription factors Nrf2 and NF-kappaB. Mol. Cell Biochem..

[76] Aziz RS, Siddiqua A, Shahzad M, Shabbir A, Naseem N (2019). Oxyresveratrol ameliorates ethanol-induced gastric ulcer via downregulation of IL-6, TNF-α, NF-ĸB, and COX-2 levels, and upregulation of TFF-2 levels. Biomed. Pharmacother..

[77] Tanaka T, Narazaki M, Kishimoto T (2014). IL-6 in inflammation, immunity, and disease. Cold Spring Harb. Perspect. Biol..

[78] Li W (2015). Protective effect of δ-amyrone against ethanol-induced gastric ulcer in mice. Immunobiology.

[79] Kobayashi EH (2016). Nrf2 suppresses macrophage inflammatory response by blocking proinflammatory cytokine transcription. Nat. Commun..

[80] Lanas A (2008). Role of nitric oxide in the gastrointestinal tract. Arthritis Res. Ther..

[81] Yu C, Mei X, Zheng Y, Xu D (2014). Gastroprotective effect of taurine zinc solid dispersions against absolute ethanol-induced gastric lesions is mediated by enhancement of antioxidant activity and endogenous PGE2 production and attenuation of NO production. Eur. J. Pharmacol..

[82] Hirahashi M (2014). Induced nitric oxide synthetase and peroxiredoxin expression in intramucosal poorly differentiated gastric cancer of young patients. Pathol. Int..

[83] Cho Y-S, Lee S-H, Kim S-K, Ahn C-B, Je J-Y (2011). Aminoethyl-chitosan inhibits LPS-induced inflammatory mediators, iNOS and COX-2 expression in RAW264.7 mouse macrophages. Process Biochem..

[84] Warzecha Z (2014). Involvement of cyclooxygenase-1 and cyclooxygenase-2 activity in the therapeutic effect of ghrelin in the course of ethanol-induced gastric ulcers in rats. J. Physiol. Pharmacol..

[85] Suryavanshi SV, Kulkarni YA (2017). NF-κβ: A potential target in the management of vascular complications of diabetes. Front. Pharmacol..

[86] Konturek PC (2010). Gastric ulcer healing and stress-lesion preventive properties of pioglitazone are attenuated in diabetic rats. J. Physiol. Pharmacol..

[87] Lv Y (2019). Increased intratumoral mast cells foster immune suppression and gastric cancer progression through TNF-α-PD-L1 pathway. J. Immunother. Cancer..

[88] Stafford JB, Marnett LJ (2008). Prostaglandin E_2_ inhibits tumor necrosis factor-alpha RNA through PKA type I. Biochem. Biophys. Res. Commun..

