# Supplementary material for: Formulation, optimization and characterization of allantoin-loaded chitosan nanoparticles to alleviate ethanol-induced gastric ulcer: in-vitro and in-vivo studies
Source: Sci Rep. 2021 Jan 26;11:2216. doi: 10.1038/s41598-021-81183-x (PMC7838192; doi:10.1038/s41598-021-81183-x)
Supplement: Supplementary file 1 — Supplementary Table S1. [file 41598_2021_81183_MOESM1_ESM.docx]

**Scientific Reports,** Formulation, Optimization and Characterization of Allantoin-Loaded Chitosan Nanoparticles to Alleviate Ethanol-Induced Gastric Ulcer: In-Vitro and In-Vivo Studies

Reham Mokhtar Aman^1,*^, Randa A. Zaghloul^2^, Marwa S. El-Dahhan^1^

*^1^Department of Pharmaceutics, Faculty of Pharmacy, Mansoura University, Mansoura, 35516, Egypt*

*^2^Department of Biochemistry, Faculty of Pharmacy, Mansoura University, Mansoura, 35516, Egypt.*

*[rehamaman@yahoo.com](mailto:rehamaman@yahoo.com)

|  | |  | **Q**  **(mg)** | **DEE**  **(%)** | **Particle size (nm)** | **ZP**  **(mV)** |
| --- | --- | --- | --- | --- | --- | --- |
| ICPs | CS concentration (X_1_) | F-value | < 0.0001^*^ | < 0.0001^*^ | < 0.0001^*^ | < 0.0001^*^ |
|  |  | Estimate coefficient | +0.4499 | +3.339 | +66.27 | +2.59 |
|  | STPP concentration (X_2_) | F-value | < 0.0001^*^ | < 0.0001^*^ | < 0.0001^*^ | 0.3970 |
|  |  | Estimate coefficient | -0.4415 | -3.933 | +56.39 | +0.1250 |
|  | CS:STPP volume ratio (X_3_) | F-value | < 0.0001^*^ | < 0.0001^*^ | < 0.0001^*^ | < 0.0001^*^ |
|  |  | Estimate coefficient | +0.2330 | +1.932 | -28.03 | +0.7458 |
|  | ALL amount (X_4_) | F-value | 0.0103^*^ | < 0.0001^*^ | < 0.0001^*^ | < 0.0001^*^ |
|  |  | Estimate coefficient | +0.0742 | -4.146 | -31.90 | +1.246 |
| ICPs interaction | CS concentration vs. STPP concentration | F-value | < 0.0001^*^ | < 0.0001^*^ | 0.1160 | < 0.0001^*^ |
|  |  | Estimate coefficient | -0.2643 | -2.609 | +4.737 | +1.171 |
|  | CS concentration vs. CS:STPP volume ratio | F-value | 0.0011^*^ | 0.0014^*^ | 0.5681 | < 0.0001^*^ |
|  |  | Estimate coefficient | +0.0981 | +0.8044 | +1.692 | +0.8417 |
|  | CS concentration vs. ALL amount | F-value | < 0.0001^*^ | < 0.0001^*^ | 0.0139^*^ | 0.1188 |
|  |  | Estimate coefficient | +0.5472 | +4.312 | -7.633 | +0.2333 |
|  | STPP concentration vs. CS:STPP volume ratio | F-value | < 0.0001^*^ | < 0.0001^*^ | < 0.0001^*^ | 0.1465 |
|  |  | Estimate coefficient | +0.2680 | +2.272 | -13.49 | -0.2167 |
|  | STPP concentration vs. ALL amount | F-value | 0.5624 | 0.0090^*^ | < 0.0001^*^ | 0.0006^*^ |
|  |  | Estimate coefficient | +0.0159 | +0.6410 | +17.61 | +0.5583 |
|  | CS:STPP volume ratio vs. ALL amount | F-value | 0.0001^*^ | 0.0014^*^ | 0.9640 | 0.0208^*^ |
|  |  | Estimate coefficient | +0.1202 | +0.8037 | -0.1333 | +0.3542 |
|  | CS concentration vs. STPP concentration vs. CS:STPP volume ratio | F-value | < 0.0001^*^ | < 0.0001^*^ | 0.5172 | 0.2493 |
|  |  | Estimate coefficient | -0.4300 | -3.812 | +1.921 | +0.1708 |
|  | CS concentration vs. STPP concentration vs. ALL amount | F-value | < 0.0001^*^ | < 0.0001^*^ | < 0.0001^*^ | 0.0001^*^ |
|  |  | Estimate coefficient | +0.2267 | +2.301 | +41.78 | -0.6292 |
|  | CS concentration vs. CS:STPP volume ratio vs. ALL amount | F-value | 0.0275^*^ | 0.0584 | 0.0797 | 1.0000 |
|  |  | Estimate coefficient | +0.0629 | +0.4523 | +5.308 | +6.06278E-16 |
|  | STPP concentration vs. CS:STPP volume ratio vs. ALL amount | F-value | 0.0015^*^ | 0.0270^*^ | 0.0018^*^ | 0.0012^*^ |
|  |  | Estimate coefficient | +0.0943 | +0.5339 | -9.962 | -0.5167 |
|  | CS concentration vs. STPP concentration vs. CS:STPP volume ratio vs. ALL amount | F-value | 0.5795 | 0.0090^*^ | 0.3857 | 0.0002^*^ |
|  |  | Estimate coefficient | +0.0152 | +0.6403 | +2.579 | -0.6125 |

**Supplementary Table S1.** Statistical analysis for the effect of ICPs and their interactions on the DMPs. *Significant at *p* < 0.05.
