# Supplementary material for: Formulation, optimization and characterization of allantoin-loaded chitosan nanoparticles to alleviate ethanol-induced gastric ulcer: in-vitro and in-vivo studies
Source: Sci Rep. 2021 Jan 26;11:2216. doi: 10.1038/s41598-021-81183-x (PMC7838192; doi:10.1038/s41598-021-81183-x)
Supplement: Supplementary file 2 — Supplementary Table S2. [file 41598_2021_81183_MOESM2_ESM.docx]

**Scientific Reports,** Formulation, Optimization and Characterization of Allantoin-Loaded Chitosan Nanoparticles to Alleviate Ethanol-Induced Gastric Ulcer: In-Vitro and In-Vivo Studies

Reham Mokhtar Aman^1,*^, Randa A. Zaghloul^2^, Marwa S. El-Dahhan^1^

*^1^Department of Pharmaceutics, Faculty of Pharmacy, Mansoura University, Mansoura, 35516, Egypt*

*^2^Department of Biochemistry, Faculty of Pharmacy, Mansoura University, Mansoura, 35516, Egypt.*

*[rehamaman@yahoo.com](mailto:rehamaman@yahoo.com)

| **Parameter** | | | | **DMPs** |
| --- | --- | --- | --- | --- |
| **Adequate precision** | **Predicted R-squared** | **Adjusted R-squared** | **R-squared** |  |
| 40.2394 | 0.9554 | 0.9709 | 0.9802 | Q (mg) |
| 42.4175 | 0.9626 | 0.9756 | 0.9834 | DEE (%) |
| 27.8406 | 0.9489 | 0.9666 | 0.9773 | Particle size (nm) |
| 22.0360 | 0.8844 | 0.9245 | 0.9486 | ZP (mV) |

**Supplementary Table S2.** Fit statistics summary for the linear regression analysis models of all the DMPs.
